# Functional and structural brain network correlates of visual hallucinations in Lewy body dementia

**DOI:** 10.1093/brain/awac094

**Published:** 2022-03-09

**Authors:** Ramtin Mehraram, Luis R Peraza, Nicholas R E Murphy, Ruth A Cromarty, Sara Graziadio, John T O’Brien, Alison Killen, Sean J Colloby, Michael Firbank, Li Su, Daniel Collerton, John Paul Taylor, Marcus Kaiser

**Affiliations:** Experimental Oto-rhino-laryngology (ExpORL) Research Group, Department of Neurosciences, KU Leuven, Leuven, Belgium; NIHR Newcastle Biomedical Research Centre, Campus for Ageing and Vitality, Newcastle upon Tyne, UK; Translational and Clinical Research Institute, Newcastle University, Campus for Ageing and Vitality, Newcastle upon Tyne, UK; Interdisciplinary Computing and Complex BioSystems (ICOS) research group, School of Computing, Newcastle University, Newcastle upon Tyne, UK; IXICO Plc, London, UK; Baylor College of Medicine, Menninger Department of Psychiatry and Behavioral Sciences, Houston, TX 77030, USA; The Menninger Clinic, Houston, TX 77035, USA; Michael E. DeBakey VA Medical Center, 2002 Holcombe Boulevard, Houston, TX 77030, USA; Translational and Clinical Research Institute, Newcastle University, Campus for Ageing and Vitality, Newcastle upon Tyne, UK; NIHR Newcastle in vitro Diagnostics Cooperative, Newcastle upon Tyne Hospitals NHS Foundation Trust, Newcastle upon Tyne, UK; Department of Psychiatry, University of Cambridge School of Medicine, Cambridge, UK; Translational and Clinical Research Institute, Newcastle University, Campus for Ageing and Vitality, Newcastle upon Tyne, UK; Translational and Clinical Research Institute, Newcastle University, Campus for Ageing and Vitality, Newcastle upon Tyne, UK; Translational and Clinical Research Institute, Newcastle University, Campus for Ageing and Vitality, Newcastle upon Tyne, UK; Department of Psychiatry, University of Cambridge School of Medicine, Cambridge, UK; Department of Neuroscience, The University of Sheffield, Sheffield, UK; Translational and Clinical Research Institute, Newcastle University, Campus for Ageing and Vitality, Newcastle upon Tyne, UK; Translational and Clinical Research Institute, Newcastle University, Campus for Ageing and Vitality, Newcastle upon Tyne, UK; Interdisciplinary Computing and Complex BioSystems (ICOS) research group, School of Computing, Newcastle University, Newcastle upon Tyne, UK; NIHR Nottingham Biomedical Research Centre, School of Medicine, University of Nottingham, Nottingham, UK; Sir Peter Mansfield Imaging Centre, School of Medicine, University of Nottingham, Nottingham, UK; Department of Functional Neurosurgery, Ruijin Hospital, Shanghai Jiao Tong University School of Medicine, Shanghai, China

**Keywords:** graph, Parkinson, EEG, MRI, diffusion

## Abstract

Visual hallucinations are a common feature of Lewy body dementia. Previous studies have shown that visual hallucinations are highly specific in differentiating Lewy body dementia from Alzheimer’s disease dementia and Alzheimer–Lewy body mixed pathology cases. Computational models propose that impairment of visual and attentional networks is aetiologically key to the manifestation of visual hallucinations symptomatology. However, there is still a lack of experimental evidence on functional and structural brain network abnormalities associated with visual hallucinations in Lewy body dementia.

We used EEG source localization and network based statistics to assess differential topographical patterns in Lewy body dementia between 25 participants with visual hallucinations and 17 participants without hallucinations. Diffusion tensor imaging was used to assess structural connectivity between thalamus, basal forebrain and cortical regions belonging to the functionally affected network component in the hallucinating group, as assessed with network based statistics. The number of white matter streamlines within the cortex and between subcortical and cortical regions was compared between hallucinating and not hallucinating groups and correlated with average EEG source connectivity of the affected subnetwork. Moreover, modular organization of the EEG source network was obtained, compared between groups and tested for correlation with structural connectivity.

Network analysis showed that compared to non-hallucinating patients, those with hallucinations feature consistent weakened connectivity within the visual ventral network, and between this network and default mode and ventral attentional networks, but not between or within attentional networks. The occipital lobe was the most functionally disconnected region. Structural analysis yielded significantly affected white matter streamlines connecting the cortical regions to the nucleus basalis of Meynert and the thalamus in hallucinating compared to not hallucinating patients. The number of streamlines in the tract between the basal forebrain and the cortex correlated with cortical functional connectivity in non-hallucinating patients, while a correlation emerged for the white matter streamlines connecting the functionally affected cortical regions in the hallucinating group.

This study proposes, for the first time, differential functional networks between hallucinating and not hallucinating Lewy body dementia patients, and provides empirical evidence for existing models of visual hallucinations. Specifically, the outcome of the present study shows that the hallucinating condition is associated with functional network segregation in Lewy body dementia and supports the involvement of the cholinergic system as proposed in the current literature.

## Teaser

Using EEG and diffusion tensor imaging, Mehraram *et al.* demonstrate that reduced structural cholinergic innervation as well as lower functional connectivity of the visual system may be key contributors to the manifestation of visual hallucinations in Lewy body dementia.

**See Zarkali and Weil (https://doi.org/10.1093/brain/awac170) for a scientific commentary on this article.**

## Introduction

Lewy body dementia (LBD) comprises dementia with Lewy bodies (DLB) and Parkinson’s disease dementia (PDD), which share similar pathology and clinical phenotype.^1,2^ Complex visual hallucinations (VH) are a clinical feature, occurring in about 80% of clinically diagnosed cases.^3–6^ Hallucinations are typically of people, animals and inanimate objects,^7,8^ tend to have short duration and may be triggered by impoverished visual conditions, such as low-lighting.^9–11^ VH were shown to have high specificity in discriminating LBD from Alzheimer’s disease and Alzheimer–Lewy body mixed pathology cases,^12–15^ as well as to be associated with poorer outcome and increased institutionalization in the patients.^11,16^ Hence, this is a topic of high interest in clinical research and an important treatment target.

A consistent picture of the pathophysiology of VH in LBD has not yet been defined. Clinical studies have suggested that generation of VH may be due to a dysfunction of multiple neurotransmitter systems, including the cholinergic system throughout visual and attentional brain networks.^17–22^ There are a number of existing models specific to Lewy body disorders. It has been suggested that a functional mismatch between the top-down [i.e. prefrontal cortex (PFC)—inferior-temporal cortex (IT) network] and bottom-up visual streams [i.e. occipital cortex (OC)—IT network] is associated with the pathological mechanisms generating VH.^3,23,24^ Evidence supporting this hypothesis includes PFC grey matter atrophy,^25^ hypometabolism within occipital and temporal areas,^26,27^ reduced activation over secondary visual areas^28^ and lower occipital GABA levels.^29^ The role of altered visual pathways in the VH phenotype was also proposed by other models, where VH are associated with defective visual and episodic memory and attentional mechanisms.^18,30^ Attentional impairment is also central in the model by Shine *et al.*^21^ who linked VH to a dysfunctional interaction between the dorsal attentional network, default mode network (DMN) and ventral attentional network (VAN); this hypothesis is supported by a functional MRI study showing reduced activation of areas within the dorsal attentional network and lower connectivity between VAN and DMN associated with lower grey matter within the insula.^22^ Moreover, other studies reported an over-activation of DMN associated with VH.^31,32^ However, these results contrast with Hepp *et al.*^33^ who failed to find any significant functional connectivity difference within the attentional networks of hallucinating and non-hallucinating Parkinson’s disease patients, although widespread reduced connectivity emerged in the first group compared to healthy controls. Outside of Lewy body specific models, but potentially still applicable, ffytche^34^ attributed a central role to hyper- or hypo- connectivity between visual and other brain regions at the time of manifestation of VH.

EEG is used in clinical research to assess communication dynamics within brain, by inferring pathological alterations of neuronal excitation and inhibition processes.^35–37^ Studies involving task-based experimental protocols reported a correlation between latency of event-related potential components and visual stimuli in LBD with VH (LBD-VH),^38–41^ while resting-state research showed lower dominant frequency in the power spectrum, lower beta (β)-band power and right temporal connectivity^42,43^ and higher delta (δ)-band and alpha (α)-band power^44^ compared to patients without hallucinations (LBD-NVH) and healthy controls. Previous studies proposed that the cholinergic system must have a primary role in the generation of VH-related functional brain alterations in LBD.^17–22^ This idea is supported by findings that include the association between integrity of the cholinergic system and EEG α-power,^45^ a restoration effect by cholinergic medication on EEG measurements returning towards normative values in patients diagnosed with DLB,^46–48^ the ability of the treatment to suppress VH^49^ and degeneration of the nucleus basalis of Meynert (NBM) in LBD-VH.^50,51^

Pathology-related network features can also be inferred by measuring structural brain properties. Diffusion tensor imaging (DTI) is reportedly an effective method to assess the structural connectome in healthy and pathological condition,^52,53^ and in combination with EEG can overcome the limitations of measuring subcortical correlates and provide a comprehensive picture of the disease-related brain alterations.^54–56^ Recent studies have reported altered white matter properties associated with LBD-VH, including the aforementioned abnormal changes of the NBM,^50^ correlation between mean diffusivity and VH severity within the right thalamus^57^ and reduced widespread structural connectivity associated with VH.^58,59^ So far, no study has investigated the association between functional and structural correlates of LBD-VH using EEG and DTI in a combined fashion.

**Figure 1:**
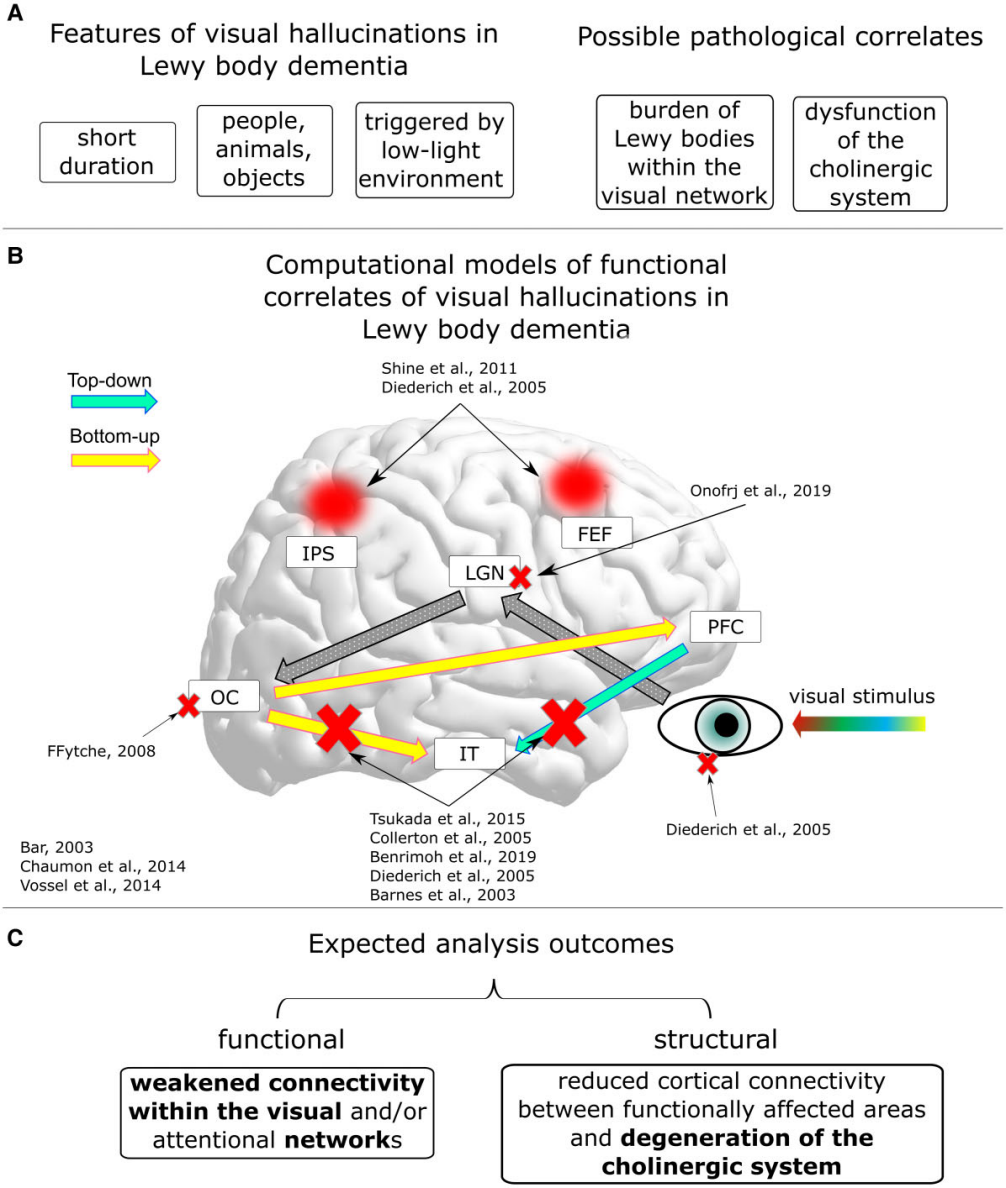
**Hypotheses of the present work.** (**A**) Key elements related to the literature of VH in LBD. (**B**) Scheme of proposed models describing the visual processing streams and hallucinations functional mechanisms in LBD. FEF = frontal eye fields; IPS = intraparietal sulcus; LGN = lateral geniculate nuclei of the thalamus. Red crosses mark disrupted connections according to the cited models; red circles mark reduced engagement of areas belonging to the dorsal attentional network according to the cited models. References shown in the figure: Shine *et al*.,^21^ Diederich *et al*.,^18^ Onofrj *et al*.,^20^ ffytche,^34^ Bar,^74^ Chaumon *et al*.,^75^ Vossel *et al*.,^76^ Tsukada *et al*.,^23^ Collerton *et al*.,^3^ Benrimoh *et al*.,^24^ and Barnes *et al*.^30^ (**C**) Expected results from the functional and structural connectivity analysis. Hypotheses confirmed by our results are highlighted in bold.

In the present work, we used EEG to assess cortical network alteration patterns associated with LBD-VH. We focused our analysis on the α-band network, as it has reportedly been associated with lower level visual and attentional processing^60–65^ and abnormal alteration of α-band rhythms has been reported in LBD against other forms of dementia and healthy condition.^66,67^ To investigate any involvement of the cholinergic system and the thalamic structure in VH-related EEG abnormalities, we used DTI to assess structural connectivity between the NBM, the thalamus and the cortical regions associated with VH in our EEG analysis, and tested its relationship with extracted EEG features. We included the thalamus due to its regulatory role on EEG oscillations^68–71^ and its reported involvement in the clinical phenotype of LBD.^20,72,73^ In line with existing models, we hypothesized that functional and structural connectivity within the visual network would be significantly affected by LBD-VH neuropathology (Fig. 1).

## Materials and methods

### Participants

Participant recruitment took place at the Newcastle Biomedical Research Centre, Newcastle University. Our sample comprised 25 LBD-VH patients (74 ± 6 years old, 11 DLB and 14 PDD) and 17 LBD-NVH patients (74 ± 6 years old, 7 DLB and 10 PDD). Groups are referred to as VH and NVH in the figures. Diagnoses were performed by two experienced clinicians according to the DLB consensus criteria^77,78^ and the diagnostic criteria for PDD.^79^ Demographic information on the participants is reported in Table 1. Global cognition was assessed through the Mini-Mental State Examination (MMSE), and the Unified Parkinson’s Disease Rating Scale part III (UPDRS-III) was used to assess motor signs. To exclude patients with too-severe cognitive impairment,^80^ participants with a MMSE score <12 were excluded from the sample, resulting in the exclusion of one PDD patient with VH with MMSE = 8. Levodopa equivalent daily dose was estimated for patients on dopaminergic medication.^81^ To exclude any association between absence of VH and cholinergic medication in the LBD-NVH group, clinical histories of those patients were checked, and no record of VH either before or after the medication onset was reported. All patients did not have other neurological or psychiatric conditions apart from dementia and written informed consent was provided by themselves or their caregivers before recruitment. This study was approved by the Northumberland Tyne and Wear NHS Trust and Newcastle ethics committee.

**Table 1:** Demographic data and clinical scores

Demographic information	LBD-VH (*n* = 25)		LBD-NVH (*n* = 17)		Statistics
Age	73.52	±6.17	74.06	±6.18	*P* = 0.949^a^
Male/female	24/1		13/4		*P* = 0.055^b^ (d.f. = 1)
DLB/PDD	11/14		7/10		*P* = 0.856^b^ (d.f. = 1)
MMSE	23.04	±3.78	24.88	±4.08	*P* = 0.067^a^
Duration of dementia, years	2.1	±3.3	4.4	±5.6	*P* = 0.253^a^
NPI hall number	1.56	±0.87	0	0	–
NPI hall frequency	2.08	±1.04	0	0	–
NPI hall severity	1.12	±0.33	0	0	–
NPI hall distress	0.92	±1.22	0	0	–
NPI hall total (frequency × severity)	2.44	±1.66	0	0	–
Complex VH (yes/no)	21/4		–		–
Duration of VH, years	2.2	±1.3	–		–
UPDRS-III	24.16	±14.27	24.59	±16.69	*P* = 0.644^a^
ACheI (yes/no)	22/3		10/6^c^		*P* = 0.076^b^ (d.f. = 2)
LEDD	540	±482	540	±497	*P* = 0.500^a^

Values in the table are reported as mean ± standard deviation. d.f. = degrees of freedom; LEDD = levodopa equivalent daily dose; UPDRS-III = Unified Parkinson’s Disease Rating Scale part III.

a Unpaired Mann–Whitney *U*-test (one-tailed for MMSE_LBD-VH_ < MMSE_LBD-NVH_).

b χ^2^ test.

c One PDD patient was on memantine.

### Experimental protocol

EEG signals were recorded in an eyes-closed resting state with a Waveguard high-density cap (ANT Neuro, Netherlands) with 128 sintered Ag/AgCl electrodes distributed according to the 10-5 system.^82^ The recording sessions lasted 2 min and took place in a dimly lit room, where participants were asked to sit at rest, keep their eyes closed and remain awake. Recordings were digitized at 1024 Hz while keeping electrode impedance <5 kΩ. The recording system used a ground electrode attached to the right clavicle, and a reference electrode in position Fz.

### EEG data preprocessing

Preprocessing of the EEG data was performed with the EEGLAB toolbox v.14^83^ on MATLAB v.9.2 (The MathWorks Inc., Natick, MA, USA, 2017). A Hamming windowed-sinc finite impulse response filter was applied with a range of 0.5 and 80 Hz, with a 50-Hz notch filter applied to remove power line noise. The obtained time-series were segmented in 2-s time epochs, and visually inspected to remove noisy or disconnected channels (respectively showing systematic bursts or flat activity over time) (number of removed channels: 16 ± 16) as well as epochs featuring sporadic artefactual activity including voltage bursts or isolated peaks (number of removed epochs: 9 ± 8). Independent component analysis (ICA) was performed using the InfoMax algorithm,^84^ with data dimensionality reduction through principal component analysis. The obtained components were visually inspected, and artefactual patterns were rejected according to established canonical classifications in the literature; these included eye blinks, heartbeat and muscular contractions (number of removed components: 36 ± 12).^85,86^ The preserved components were projected back to the time-series domain.

### MRI: recording and preprocessing

MRI recordings were performed on a 3-T Philips Intera Achieva scanner with magnetization prepared rapid gradient echo sequence, sagittal acquisition, echo time 4.6 ms, repetition time 8.3 ms, inversion time 1250 ms, flip angle = 8°, SENSE factor = 2, in-plane field of view 240 × 240 mm^2^ with a slice thickness of 1.0 mm, yielding a voxel size of 1.0 × 1.0 × 1.0 mm^3^.^45,87^ Preprocessing and segmentation of acquired T_1_-weighted images were performed using FreeSurfer (v. 5.1, http://surfer.nmr.mgh.harvard.edu/).^88,89^ The automated processing pipeline included intensity non-uniformity correction, Talairach registration, removal of non-brain tissue (i.e. skull stripping), white matter and subcortical grey matter segmentation, tessellation of the grey–white matter boundary and surface deformation following grey matter–CSF intensity gradients for optimal placing of grey–white matter and grey matter–CSF borders. Modelling of the cortical surface was followed by surface inflation, transformation to spherical atlas and parcellation into 148 regions according to the Destrieux atlas.^90^ This parcellation was preferred due to its high resolution and inclusion of only cortical regions. Resulting images from each processing step were visually inspected and, where required, manually corrected to ensure accurate segmentation.^91,92^

DTI recordings were performed with a two-dimensional spin-echo, echo planar imaging diffusion-weighted sequence with 59 slices: repetition time = 6100 ms; echo time = 70 ms; flip angle = 90°; field of view = 270 × 270 mm; pixel size= 2.1 × 2.1 mm and slice thickness = 2.1 mm. Images were diffusion weighted along 64 uniformly distributed directions (diffusion contrast *b* = 1000 s·mm^−2^), and six acquisitions did not have any diffusion weight applied (*b* = 0 s·mm^−2^).^93^ DTI recordings were then corrected for eddy current distortion, movement and motion-induced signal dropout using the ‘eddy' package.^94,95^ Since different nuisance profiles may have potentially affected our analysis, we made sure that there was no difference in head movement between groups by comparing the average volumetric root mean squares of the head movement with a Mann–Whitney *U*-test, which yielded an insignificant outcome (*U* = 545, *P* = 0.858).

### Cortical source localization

Cortical source reconstruction from EEG signals was obtained with the standardized low resolution brain electromagnetic tomography (sLORETA)^96^ implemented in the Brainstorm toolbox for MATLAB.^97^ Previous studies proved a higher accuracy of this method compared with other existing non-parametric methods in the literature,^98^ and its suitability for connectivity analysis.^99^ Due to unavailability of digitized sensor localization, we manually coregistered the EEG sensors distribution over the scalp for each participant’s MRI using the Brainstorm toolbox.^100^ We used the boundary element method as implemented in OpenMEEG^101,102^ to build the head models based on the individual T_1_-weighted MRI data. Noise covariance was set to an identity matrix, reconstruction of cortical sources was performed assuming a normal dipole orientation with respect to cortical surface, and the obtained source-domain time-series were averaged within each cortical region after flipping dipoles with opposite signs to match the main orientation. Validation of the implemented source localization pipeline was made using EEG data recorded during a task-based paradigm from subjects of the same cohort and is reported in the Supplementary material.

### Weighted phase lag index and modularity

Connectivity between cortical sources was measured with the weighted phase lag index (WPLI),^103^ which is a measure of synchronization between any couple of signals across time. Its mathematical formulation is:

(1) $$WPLI=\frac{\left|\left\langle Im(X)sign[\Delta \phi (t_{k})]\right\rangle \right|}{\left|\left\langle Im(X)\right\rangle \right|}$$

where *X* is the cross-spectrum between the signals, *Im*(*X*) is its imaginary part, Δ*ϕ* is the phase difference between the signals, *t_k_* is the time step with *k* = 1, 2, …, *N* and *sign* is the signum function. Connectivity values span between 0 (lack of connectivity) and 1 (full connectivity). The WPLI is insensitive to almost-zero- and zero-lagging synchronization between signals, i.e. Δ*ϕ* ≈ / = 0, typically reflecting volume conduction, which is a common issue in EEG recordings.^104^ Connectivity was computed using functions included in the Fieldtrip toolbox.^105^ Specifically, EEG source time-series were first transformed into the frequency domain using Windowed Fourier Transform (3–10 cycles adaptive window width, 0.5 frequency step) and WPLI within the α-band (8–13.5 Hz) was computed. WPLI values were averaged across time and frequency bins, resulting in one connectivity matrix per subject. Weighted modularity (*Q_w_*) was also computed from each connectivity matrix as implemented in the Brain Connectivity Toolbox (BCT) for MATLAB,^106^ on the basis of the following formulation:^107^

(2) $$Q_{w}=l^{(w)-1}\underset{i,j\in N}{\sum }\;[w_{ij}-l^{(w)-1}(K_{i}K_{j})]\delta_{m_{i}m_{j}}$$

where *m_i_* is the module containing the *i*-node, *w_ij_* is the connection weight between *i*-node and *j*-node, *l* is the number of edges, *K_i_* is the *i*-node strength, and *δ* is the Kronecker delta function; strength of a *i*-node is defined as the sum of the weights of the edges connecting to that node.^106,108^ Higher values of *Q_w_* indicate stronger network segregation.

For completeness and exploratory purpose, these measures were also extracted and the subsequent analyses performed for the theta (4.6–7 Hz) and beta (15–20 Hz) frequency bands, as described in the Supplementary material.

### EEG-network statistical analysis

For the statistical tests we used MATLAB and the Statistical Package for the Social Sciences v.24. Differential topographical network patterns between LBD-VH and LBD-NVH were obtained using the Network Based Statistics (NBS) toolbox.^109^ All connections were first tested against the null hypothesis that mean connectivity is equal between groups. A test statistic threshold (*t_th_*) was chosen, and those connections whose *t*-values overcame *t_th_* were candidates to form a significant network component. The size of the detected significant component, i.e. sum of individual connection *t*-values, was computed and data were permuted between groups. This process iterated 5000 times, and the largest component sizes were recorded. The family wise error rate-corrected *P*-value for each component was obtained by computing the ratio between number of iterations at which the largest component was of the same size of the current component or greater and total number of permutations. The choice of *t_th_* is arbitrary, and we chose to push the values towards the strictest thresholding to obtain as local differential topographies as possible. This resulted in performing two *F*-tests at, respectively, *t_th_* = 14 and *t_th_* = 15.4, controlling for the MMSE score; network differential components were deemed significant at *P* < 0.05, and were visualized with the BrainNet Viewer.^110^ We then computed the average connectivity strength across the NBS connections (WPLI_NBS_) and compared it between groups with a Mann–Whitney U-test (*P* < 0.05).

We also computed the node strengths (*K*) within the NBS component and compared them between groups by performing a Wilks’ Lambda multivariate test (*P* < 0.05) followed by *post hoc* Mann–Whitney U-tests (*P* < 0.05, Holm–Bonferroni corrected for number of NBS nodes). *K* computation was preceded by dividing connection weights within the NBS component by the maximum WPLI value^111^ to attenuate any connectivity strength bias potentially affecting the network measure.

We compared the modularity between groups with analysis of covariance test corrected for MMSE (*P* < 0.05), while the modular distribution was obtained for both groups using routines implemented in the BCT. For each EEG network, the optimal community structure was obtained and each node was assigned to a module; an agreement matrix was obtained for each group, where each element indicated the frequency within the group for which every couple of nodes belonged to the same module; the elements of the agreement matrix were divided by the number of networks; a threshold was applied on the obtained matrix preserving probabilities above 64% and eventually, a consensus matrix was computed.^112^ The obtained modular distribution was visualized with the BrainNet Viewer by identifying each module with a colour. For each group, proportion of within-module NBS connections was computed as a ratio between number of within-module NBS connections and total number of NBS connections.

For topographical visualization, network nodes were defined as mass centroids across head-model vertices within each corresponding region. Coordinates of network nodes in the Montreal Neurological Institute (MNI) reference space are reported in Supplementary Table 1.

### White matter fibres tractography and statistical analysis

**Figure 2:**
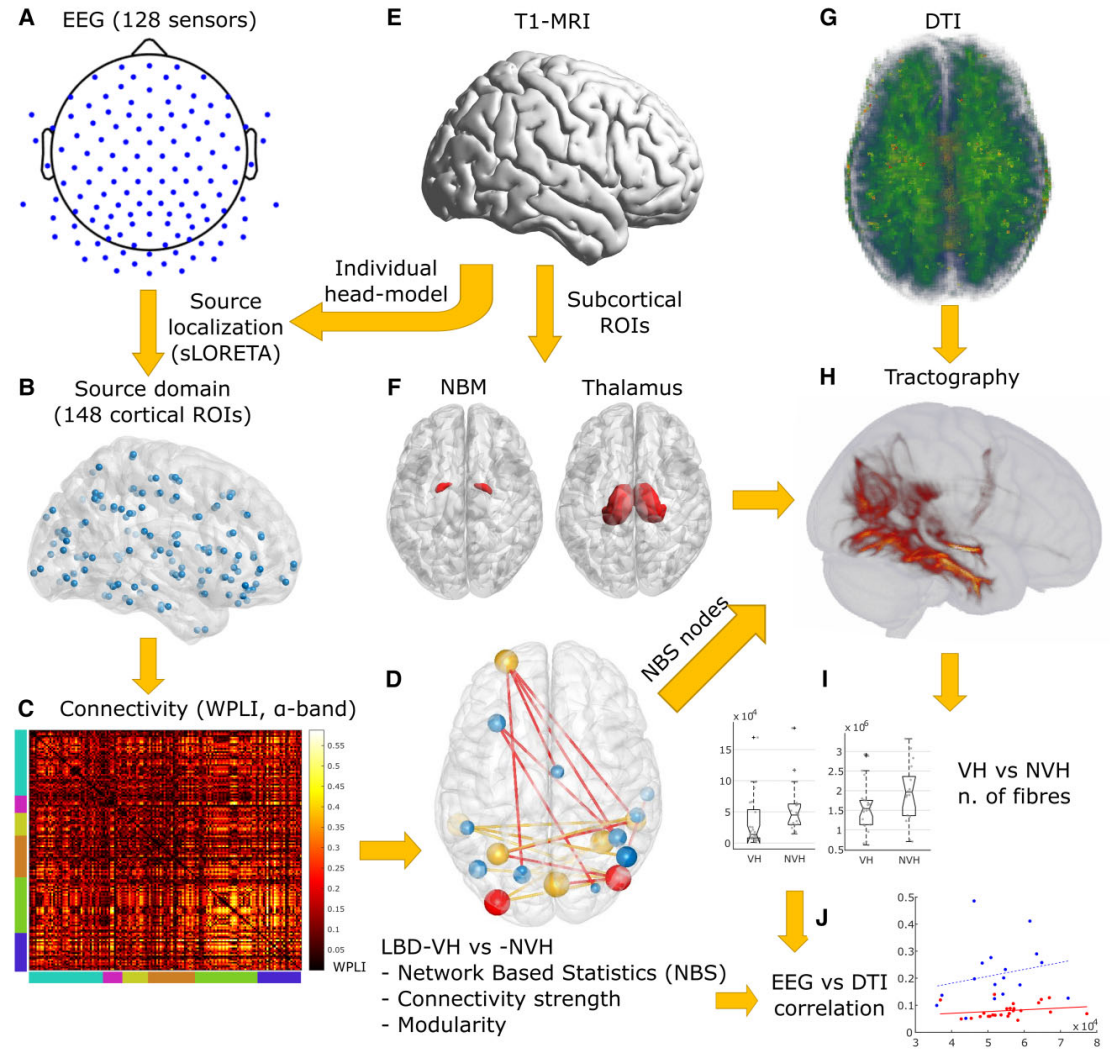
**Methodological workflow.** (**A**) EEG-electrode distribution over the scalp. (**B**) Network nodes distribution within the cortex. (**C**) Example of a source-domain connectivity matrix in the α-band network of an NVH subject. Node distribution by regions is showed by bottom and left bar colours. From *left* to *right*: teal = frontal; magenta = insula; yellow = cingulate; orange = temporal; green = parietal; blue = occipital. (**D**) Output of NBS (for a description see Fig. 3). (**E**) Image of standard-MRI (Colin27). (**F**) NBM and thalamus regions of interest (ROIs) in the MNI space. (**G**) Example of a functional anisotropy map on a VH subject, projected to the MNI space. (**H**) Tractography output of a VH subject performed between NBS-detected and subcortical region of interest. (**I**) Distribution of number of streamlines in the thalamus-cortex and NBM-cortex white matter tracts. (**J**) WM_NBS_-WPLI_NBS_ distribution (for a description see Fig. 6).

To investigate whether any correlation exists between functional and structural network feature alterations associated with VH, DTI data were used to assess white matter connectivity within the functionally affected cortical regions (WM_NBS_) as well as between the cortex, the thalamus and the basal forebrain. A region of interest corresponding to the NBM (Ch4 cellular group) in the MNI space was generated with the Statistical Parametric Mapping Anatomy Toolbox for MATLAB,^113^ while the thalamus MNI region of interest was based on the Oxford thalamic connectivity atlas^114^ included in the FMRIB Software Library.^115^ To transform the subcortical region of interest to the subject space we used affine and non-linear transformations implemented in the Advanced Normalization Tools software.^116^ Cortical region of interest were defined as volume masks of the NBS-detected regions and transformed from the subject space to the diffusion space. To this purpose, a linear transformation matrix for each subject was generated with the FLIRT package^117^ using as origin and target respectively a brain-extracted T_1_ MRI image and a functional anisotropy map. The subcortical region of interest as defined in the MNI space are shown in Fig. 2.

The tractography pipeline was implemented with the FMRIB’s Diffusion Toolbox.^115^ We first assessed the local probability distribution of fibre direction at each voxel with automatic detection of number of fibres per voxel.^118,119^ A probabilistic tractography algorithm^118,119^ was then used to track white matter streamlines connecting the ROI. Connection probability between voxels was estimated as proportion of connecting fibres, over 5000 sampled fibres per voxel. For each network, the tractography resulted in a structural connectivity matrix of dimension *n* + 2 (seed masks) × *n* + 2 (target masks) streamlines, where *n* was the number of NBS nodes. Since streamline directionality could not be assessed, the connectivity matrices were symmetrized by replacing each (*i*,*j*)-element with the average between itself and the (*j*,*i*)-element.^120^

We tested whether connectivity of WM_NBS_, cortex-NBM and cortex-thalamus, i.e. the number of streamlines, was affected in LBD-VH compared to LBD-NVH (Mann–Whitney U-test, one-tailed, *P* < 0.05, three tests). We also investigated for any association between structural and functional alterations by performing Spearman rank correlation tests (*P* < 0.05, one-tailed) between the EEG features, i.e. WPLI_NBS_ (right-tailed) and *Q_w_* (left-tailed), and, respectively, the average structural connectivity of WM_NBS_ and total number of white matter streamlines connecting the cortical regions to the thalamus and NBM, respectively, for both LBD-VH and LBD-NVH groups (six tests for each EEG metric).

### Data availability

The data that support the findings of this study are available from the senior authors J.-P.T. and M.K., on reasonable request by qualified researchers.

## Results

### Demographic data

Demographic information and clinical score comparisons between groups are reported in Table 1. The participant cohort comprised DLB and PDD patients without VH as well as patients with distributed levels of VH frequency and severity, based on the Neuropsychiatric Inventory (NPI) subscale test for hallucinations; groups were matched for diagnosis, age and sex. MMSE was higher in the LBD-NVH group, with a trend towards significance, hence the NBS test and comparisons of modularity (*Q_w_*) between groups were corrected for MMSE score to exclude any confounding effect of cognition given that this is a risk factor for VH.^8,9^ Most participants (22 LBD-VH and 10 LBD-NVH) were on cholinergic medication and one LBD-NVH patient was on memantine; the percentage of patients on medication was not different across groups. UPDRS-III scores and Levodopa equivalent daily dose were not significantly different between groups.

### EEG-network topographical differences

**Figure 3:**
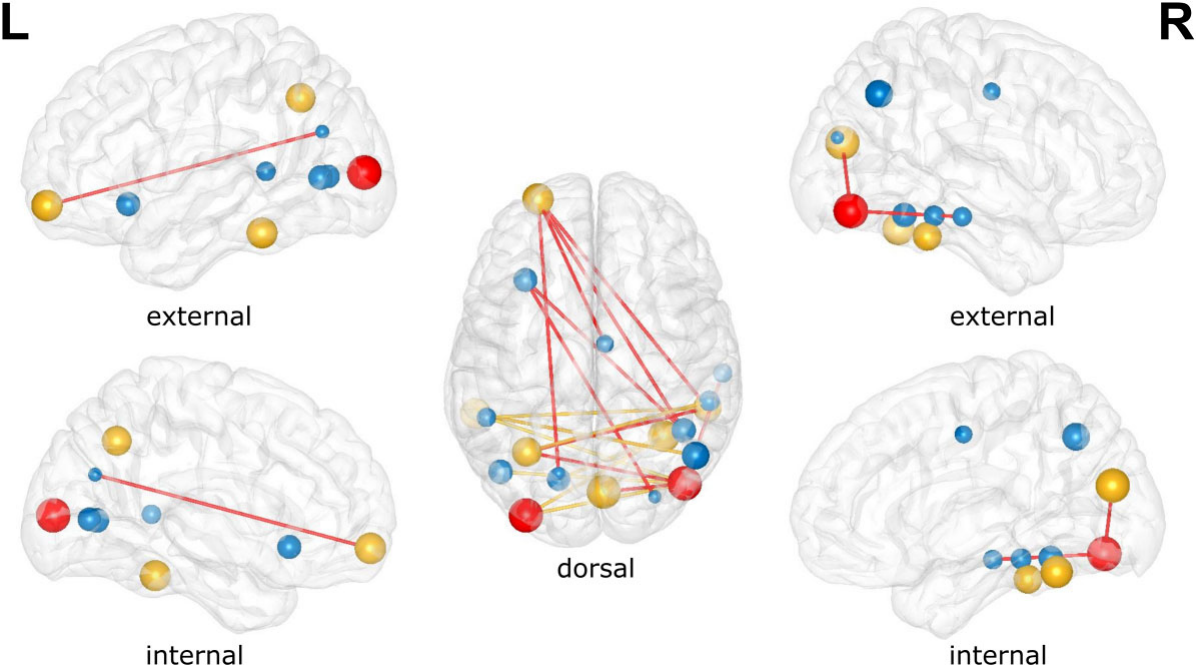
**Outcome of NBS analysis.** All edges represent the differential topography between VH and NVH obtained with *t_th_* = 14. Red edges are obtained with *t_th_* = 15.4. Non-blue nodes have significantly different strength between groups (*P* < 0.05). Red nodes survive Holm–Bonferroni correction; these comprise the right inferior-occipital gyrus and sulcus and left middle occipital sulcus and lunatus sulcus. Sphere size is inversely proportional to node’s corresponding *P*-value from the node strength test.

**Figure 4:**
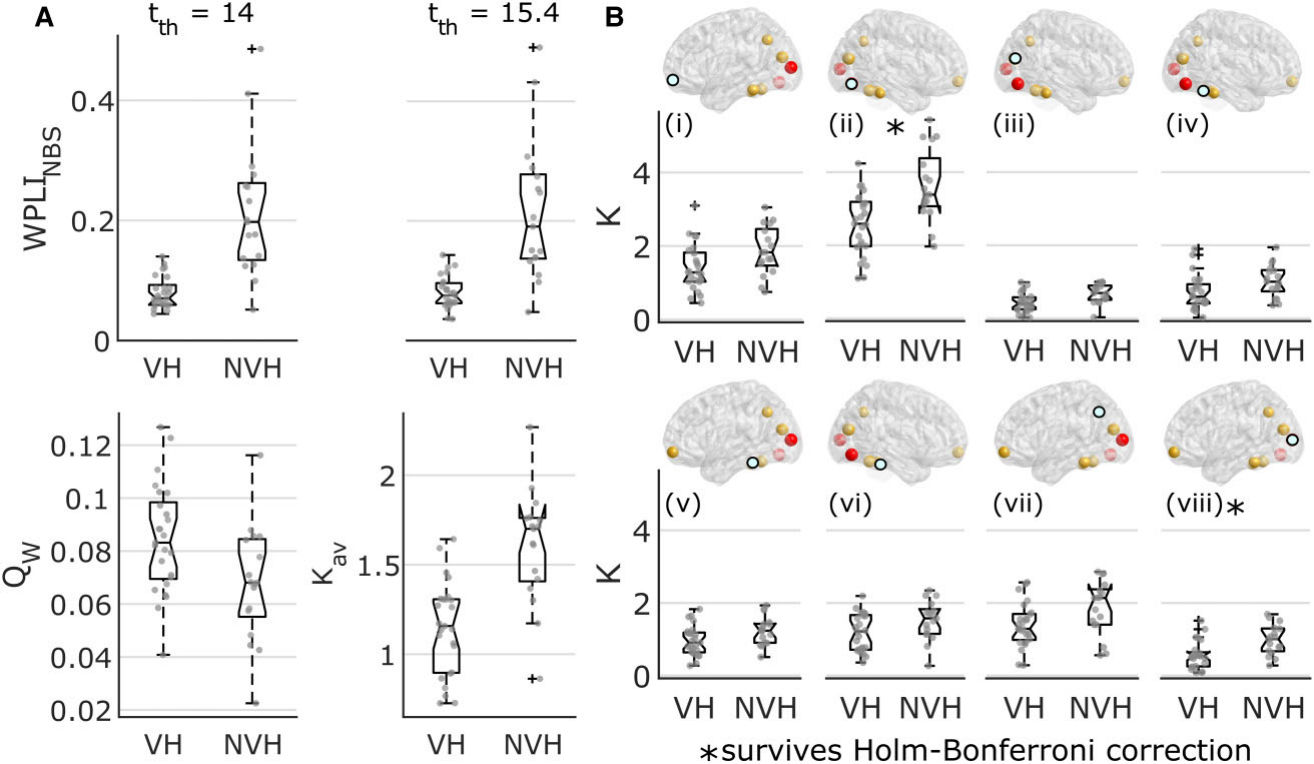
**Results from network strength analysis.** (**A**) Distribution across groups for the average connectivity within the NBS component at two different statistical thresholds, modularity, and average node strength at the lowest statistical threshold (*t_th_* = 14); the VH group showed lower connectivity strength (WPLI) and higher network segregation (*Q_w_*) compared to the NVH group (Mann–Whitney U-tests, *P* < 0.05). *WPLI_NBS_* = average WPLI within the NBS component; *Q_w_* = weighted modularity. (**B**) Individual node strength (*K*) distributions across groups of those nodes for which Mann–Whitney U comparison tests were significant (*P* < 0.05); node strengths were lower in the VH group compared to NVH; corresponding brain node for each figure is highlighted in teal; from the *top left* to the *bottom right* images: (**i**) left frontomarginal gyrus and sulcus; (**ii**) right inferior-occipital gyrus and sulcus; (**iii**) right cuneus; (**iv**) right lateral occipito-temporal gyrus; (**v**) left inferior-temporal gyrus; (**vi**) right inferior-temporal gyrus; (**vii**) left intraparietal sulcus; and (**viii**) left middle occipital sulcus and lunatus sulcus. In the box plots, whiskers extend to the most extreme data points not considered outliers. *Test survived Holm–Bonferroni correction. *K* = node strength.

Results of the NBS analysis are shown in Fig. 3 and correspond to the altered subnetwork within the α-band between LBD-VH and LBD-NVH groups. Comparison between groups at *t_th_* = 14 yielded one significant component comprising 19 nodes and 18 edges (*P* = 0.032), which included areas belonging to the OC, left and right IT, the PFC, the middle-posterior cingulate and anterior insula. Comparison between groups of the average connectivity across the edges comprised in the NBS component (WPLI_NBS_) and average strength across the network nodes within the NBS component (*K_av_*) resulted in weaker connectivity in LBD-VH compared to LBD-NVH (Fig. 4). The subnetwork produced at *t_th_* = 15.4 comprised a lower number of connections with PFC, insula, cingulate, between OC and right IT and between right IT and the left intraparietal sulcus.

Before testing individual node strength differences between groups, we performed a multivariate test, which yielded a significant result (*F*(18, 23) = 2.46, *P* = 0.022). Eight out of 19 node strengths were significantly lower in LBD-VH compared to LBD-NVH as shown in Fig. 4, and were marked in yellow or red in Fig. 3, but only the regions comprising the right inferior-occipital gyrus and sulcus (*P* = 0.0014) and left middle occipital sulcus and lunatus sulcus (*P* = 0.0016) survived Holm–Bonferroni correction.

**Figure 5:**
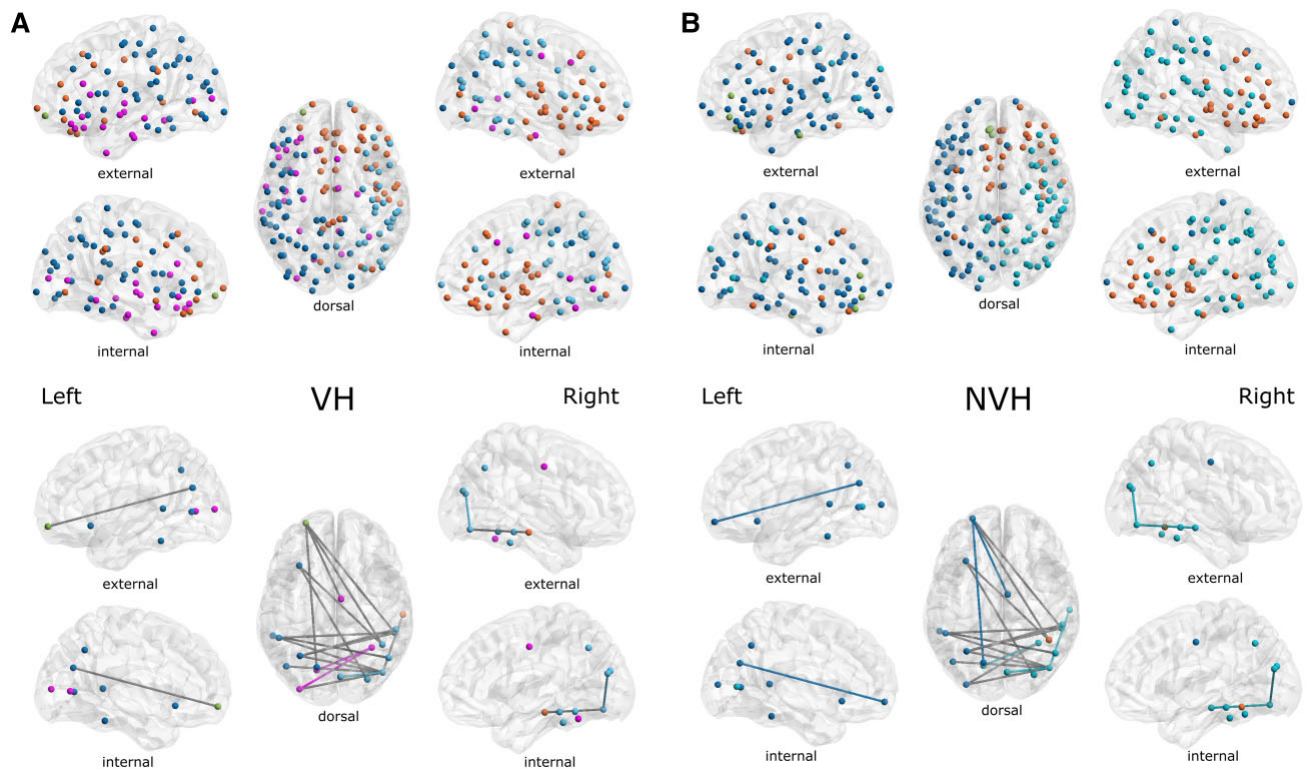
**Modular distributions.** *Top*: Community structure of the network nodes. *Bottom*: Modular distribution of the NBS edges, where a coloured edge is a connection within the module of its colour in the corresponding top figure, and grey edges are between-module connections. Modular distribution in LBD-VH was more disrupted and showed higher number of modules when compared to LBD-NVH. (**A**) LBD-VH and (**B**) LBD-NVH.

Modular organization of the network and the NBS-detected subnetwork are shown in Fig. 5. We found four modules in LBD-VH group, one including right and central-frontal regions and right temporal pole, one comprising left frontal, parietal and occipital regions and one comprising left frontal and temporal regions; one node in the left prefrontal lobe did not belong to any of the modules. The LBD-NVH network comprised three modules over the left hemisphere, the right prefrontal region and the right temporal-parietal-occipital areas; one further module only comprised three nodes within the left prefrontal region and one in the left temporal lobe. In the LBD-NVH group, 27.78% of NBS-detected edges were connecting nodes belonging to the same module, whilst in LBD-VH that was the case for 16.67%. Connections that connected within-module nodes in one group and between-module nodes in the other, or vice versa, included two edges connecting PFC to OC and cingulate, and two connections between the right temporal cortex and the right and left OC, respectively. Modularity (*Q_w_*) was significantly higher in LBD-VH compared with LBD-NVH (*F*(1, 39) = 6.06, *P* = 0.018).

As hypothesized, these results were specific to the α-band network, as also described in the Supplementary material.

### Structural versus functional connectivity

**Figure 6:**
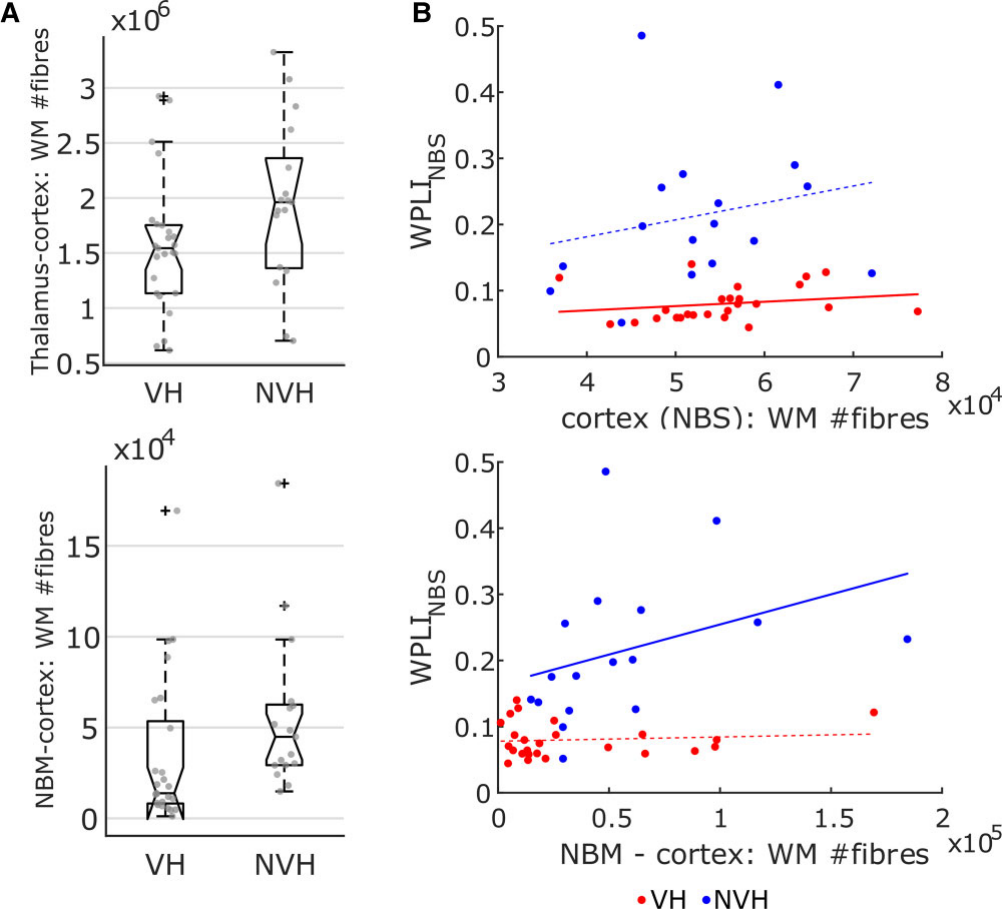
**Results from the DTI analysis.** (**A**) Distribution across groups for the number of streamlines for those tracts that yielded significant results (Mann–Whitney *U*-test, *P* < 0.05). Number of white matter (WM) streamlines was lower in VH compared to NVH for the thalamus-cortex and NBM-cortex tracts. In the box plots, whiskers extend to the most extreme data points not considered outliers. (**B**) White matter streamline count versus EEG-metric distributions for which Spearman’s rank correlation tests were significant for any group (*P* < 0.05); thicker linear regression lines represent significant correlations; a positive correlation between white matter streamline count and functional connectivity emerged in the VH group for the within-cortex tract, and in the NVH group for the NBM-cortex tract. WPLI_NBS_ = average WPLI within the NBS component; red dots = VH; blue dots = NVH.

Comparison of the number of white matter streamlines between groups produced a significant outcome for the NBM-cortex and thalamus-cortex tracts, but not for WM_NBS_. Specifically, connectivity between the thalamus and NBS-regions was significantly lower in LBD-VH compared to LBD-NVH (*U* = 457, *P* = 0.02), and the NBM-cortex streamlines were significantly less in LBD-VH, as shown in Fig. 6A (*U* = 433, *P* = 0.004); both tests survived Holm–Bonferroni correction. Significant correlations were found between WM_NBS_ and WPLI_NBS_ in LBD-VH (*ρ* = 0.42, *P* = 0.019, not surviving Holm–Bonferroni correction), as well as between number of streamlines in the NBM-cortex tract and WPLI_NBS_ in the LBD-NVH group (*ρ* = 0.58, *P* = 0.008, surviving Holm–Bonferroni correction), but no correlation with EEG connectivity emerged for the thalamus-cortex tract in either group, nor between structural connectivity and *Q_w_*. WM-WPLI_NBS_ trends are shown in Fig. 6B.

## Discussion

We investigated EEG source network abnormalities during the resting state and their correlation with structural features associated with LBD-VH. As hypothesized, we found that brain visual networks showed altered properties in the LBD-VH group compared to LBD-NVH, and we found an association with alteration of the cholinergic projections of the NBM.

### The visual ventral network is disconnected in LBD-VH

As detected with NBS, functional connectivity within the α-band network between OC, IT and PFC was reduced in LBD-VH compared to LBD-NVH. The obtained differential topography agrees with the Perception and Attention Deficit model^3^ and the computational model by Tsukada and colleagues;^23^ according to these models, VH in LBD are driven by a mismatch between the top-down, i.e. PFC to IT, and bottom-up streams, i.e. OC to IT. Notably, connectivity with multiple areas within the OC was reduced in LBD-VH, in line with reported occipital functional changes in Lewy body disease hallucinators including lower GABA level^29^ and glucose metabolism.^26,27,121^ It is possible that OC functional alteration in LBD-VH propagates along the visual ventral network and affects the IT lobes, whose functional strength was also significantly reduced (*P* < 0.05), although this observation did not survive multiple comparison correction. PFC-IT edges were also weakened, probably reflecting dysfunctions of the top-down visual stream as predicted by the models.^3,23^ Specifically, PFC regions involved in the affected network comprised the left frontomarginal sulcus and gyrus, which is in the inferior part of the frontal pole, continuous to the orbital gyrus,^90^ limiting the frontopolar and orbital regions.^122^ According to existing visual perception models, a sparse version of visual information is projected from OC to PFC,^74^ through either dorsal visual network^123^ or the thalamus,^124^ so that the orbito-frontal cortex can generate a predictive template when this limited visual information stimulus is congruent with information stored in memory.^75^ This process rapidly takes place before the stimulus goes through the bottom-up system, i.e. from OC towards IT,^125^ which is where the two streams match and an internal image, or hallucination, is generated.^74,126^ Lateralization towards the left hemisphere is in line with reported evidence of role of the left PFC for the retrieval of internal imagery.^127–129^ The involvement of the frontal areas was also reported in another functional MRI study using dynamic casual modelling, where the authors could successfully simulate association between face recognition, activation of frontal areas and effective information flow along the visual ventral network.^130^ Our findings may be considered as empirical evidence of the PAD and similar models, suggesting that in LBD-VH the information flow stream is also affected in the resting state, possibly reflecting an alteration of trait liability to VH.

With the NBS analysis we also found reduced functional interhemispheric connectivity. Association between this phenomenon and auditory hallucinations was reported for schizophrenia^131–133^; this is the first time that a similar differential topographical pattern in LBD-VH has been reported. An EEG study showed that coherence between the left and the right occipitotemporal areas early in time is positively correlated with successful object recognition, with the authors suggesting that interhemispheric information flow during visual perception is probably ‘the first gate of an active attention system'.^134^ The NBS topography resembled an interhemispheric pattern between occipitotemporal areas, suggesting that early functional processes associated with active perception might be impaired in LBD-VH, and this also emerges in the resting state. From a pathological perspective, reduced node strength within the IT might be associated with higher burden of Lewy bodies within this region, which has previously been associated with VH.^19^

### Interaction between attentional and visual networks is weakened in LBD-VH

We found that connectivity between visual areas and the right middle-posterior cingulate cortex, left anterior insula and left temporal superior sulcus, was weakened in LBD-VH. The middle-posterior cingulate is reportedly part of the DMN,^135^ while the anterior insula and temporal superior sulcus are part of the VAN.^76,136,137^ However, in the NBS component, no alteration emerged within or between these networks. This result is only partially consistent with the models proposing a role of attentional networks in the VH trait,^18,21^ which are supported by empirical evidence showing an over-activation of DMN and VAN together with a faulty engagement of dorsal attentional network associated with image misperception.^22,31,32^ We posit that this apparent contrast in results could stem from the use of a different methodological approach. In the supportive studies, regions of interest were chosen based on a prior hypothesis, and the experimental condition consisted in task-based paradigms; in contrast, we focused on affected connectivity patterns in the resting state, using a data-driven approach, i.e. NBS.^109^ We speculate that if any affected interaction between and within attentional and default mode networks in LBD-VH exists, it may not be as prominent as in the visual network and would not emerge with our strategy. On the other hand, our results indicate that the faulty interaction between attentional networks and DMN in LBD-VH may be mediated by the visual streams. Our findings are also consistent with previous research showing that grey matter of the anterior insula and hypometabolism within the middle cingulate correlate with the intensity of VH in Alzheimer’s disease dementia and Alzheimer–Lewy body mixed pathology condition.^138^

### The functional network is more segregated in LBD-VH compared to LBD-NVH

Modular network organization has been shown to be crucial for visual perception processing^139–141^ as also described in computational models.^74,75,125,142–144^ We provide for the first time evidence of functional network segregation and modular disruption in LBD-VH associated with impairment of long-range connections. Although the latter has been reported previously when comparing DLB with Alzheimer’s disease dementia,^2,67^ no direct association with VH has been reported until now. On the basis of the NBS topographies and the persistence of affected connections to PFC, even when setting a stricter NBS-*t_th_*, we theorized that the affected top-down projections may be among the drivers of the network segregation in LBD-VH. Overall, our analysis showed that visual-related functional subnetwork properties are potentially informative for the correct diagnosis of LBD against Alzheimer’s disease dementia and mixed pathology cases.

### Alteration of cholinergic pathways is associated with EEG-network abnormalities in LBD-VH

We found higher degeneration of white matter streamlines between the NBM and the NBS-detected regions in LBD-VH compared to LBD-NVH. A similar result was also reported in a study with Parkinson’s disease patients, where higher mean diffusivity was detected in hallucinating compared to non-hallucinating patients within the tracts connecting the NBM to occipital and parietal areas.^50^ The authors suggested that such projections might have a key role in the aetiology of VH in Parkinson’s disease. Interestingly, we also found a correlation between cortical functional connectivity and number of NBM-cortex white matter fibres in LBD-NVH group, but not in LBD-VH. Disrupted structure-function coupling has been reported in other conditions including bipolar disorder^145^ and migraine,^146^ but not in LBD-VH. Our results concur with the idea of a structure-function coupling as a physiological phenomenon,^54,147–149^ which is probably altered in pathological condition. Although both participant groups included LBD patients, the ones who did not develop VH might still present some intact physiological features, specifically associated with the cholinergic system. These might be more severely affected in LBD-VH, leading to the observed disrupted structural-functional correlation. In fact, we speculate that hallucinating and non-hallucinating condition are different phenotypes of the same disease, the first being driven by strategic cholinergic loss and the latter being more associated with a global cortical pathologic change.^16,150^

Structural connectivity between the thalamus and cortical regions of interest was also significantly affected in LBD-VH compared to LBD-NVH. This outcome concurs with a previous study where correlation between mean diffusivity within the right thalamic subregion and NPI hallucination score was reported in DLB.^57^ Degeneration of white matter tracts connecting the thalamus to the OC associated with VH and visual dysfunction were also reported in more recent studies.^59,151^ However, streamlines’ count within this tract did not correlate with any EEG measure; in contrast there was a positive correlation between WM_NBS_ in the LBD-VH group and WPLI_NBS_. We speculate that this latter finding may reflect the fact that VH-related pathological mechanisms may equally affect functional and structural network properties in the cortex in LBD patients.

### Limitations

Our work presents some limitations.

Although the gender distribution was similar, we found a trend towards significance in the between-group comparison. We could not correct the results for gender, as the number of female participants was too low. Since there is no evidence in literature of gender effect on VH-related pathological and functional processes, it was assumed that results were not significantly affected by any gender imbalance.

Most patients in this study were taking cholinesterase inhibitors, which may have partially restored the EEG-network properties towards normative values in the LBD-VH group,^47,48^ making group differences less distinct. Nevertheless, we found significant differences between groups. With an exploratory purpose, we additionally performed the NBS also including the medication state as a nuisance covariate; as a result, we obtained a network component resembling the one detected within our main analysis, although with a lower number of connections, possibly due to the low statistical power originating from the low number of patients not on medication. This additional analysis is reported in Supplementary material.

A methodological limitation lies in the fact that both WPLI and white matter streamline count could not provide any information on directionality of the connections. Future studies will be needed to assess whether any VH-related alteration exists in the direction of information flow between brain regions.

Concerning the EEG-network analysis, NBS threshold remains an arbitrary choice.^109^ The purpose of our research was to investigate for localized differential network properties; hence we chose conservative *t_th_* values. However, it is possible that sensitivity of NBS may vary depending on the choice of lower thresholds, producing either larger network components or no significance at all.

A potential limitation of our analysis lies in the choice of the MMSE as a cognition-related behavioural score. This score reportedly shows low sensitivity in characterizing the cognitive phenotype of patient groups, although this is mainly the case in mild cognitive impairment.^152^ Our choice was aimed to include a measure of global cognition, as focusing on cognitive subdomains was beyond the scope of our work. To address any potential concern, we also performed the NBS analysis correcting for the Cambridge Cognition Examination subdomain scores, and we detected a subnetwork that resembled the one we reported in the Results section, confirming the robustness of our results. This additional analysis is reported in the Supplementary material.

Furthermore, we choose not to investigate subtypes of VH, grouping together patients with simple and complex hallucinations, with the first being only four. Surprisingly, WPLI_NBS_ within the LBD-VH group did not show any outlier, as shown in Fig. 4. However, previous work has suggested that simple and complex hallucinations may be associated with different pathological mechanisms and excluded simple hallucinations from the LBD-VH group.^29,31,153,154^ Due to our limited sample size, we could not investigate the extent to which simple hallucinations influence functional connectivity as compared to complex hallucinations.

Although we included the thalamus in our white matter fibre tracking, we focused on the cholinergic projections towards the cerebral cortex. The majority of cholinergic innervations towards the thalamus reportedly originate from the pedunculopontine tegmentum and latero-dorsal tegmentum;^155,156^ their possible association with visual-related alterations could be investigated in future studies.

## Conclusion

In the present study, we showed that LBD features specific EEG functional network properties depending on whether patients have developed VH or not. For the first time, we demonstrate a differential topography between LBD-VH and LBD-NVH without any prior hypothesis, which agreed with models of VH. Visual processing streams and, specifically, the OC, were affected in LBD-VH compared to LBD-NVH, although an affected interaction between the ventral visual network and DMN/VAN also emerged. Modular organization of EEG source network within both top-down and bottom-up visual streams is more disrupted in LBD-VH. Our study also provides evidence of an association between disruption of the cholinergic system and functional connectivity abnormalities in LBD-VH. The results of our work validate EEG and the use of multiple recording modalities as effective tools to investigate pathological insights and treatment strategies associated with LBD and, specifically, VH.

## Supplementary Material

awac094_Supplementary_Data

## Abbreviations

DLB: dementia with Lewy bodies
DMN: default mode network
DTI: diffusion tensor imaging
IT: inferior-temporal cortex
K: node strength
LBD: Lewy body dementia
LBD-VH/NVH: Lewy body dementia with/without visual hallucinations
MMSE: Mini-Mental State Examination
NBM: nucleus basalis of Meynert
NBS: network-based statistics
NPI: Neuropsychiatric Inventory
OC: occipital cortex
PDD: Parkinson’s disease dementia
PFC: prefrontal cortex
VAN: ventral attentional network
VH: visual hallucinations
WPLI: weighted phase lag index

## References

[1] McKeith I . Dementia with Lewy bodies and Parkinson’s disease with dementia: Where two worlds collide. Pract Neurol. 2007;7:374–382. 18024777 10.1136/jnnp.2007.134163

[2] Mehraram R , Kaiser M, Cromarty R, et al Weighted network measures reveal differences between dementia types: An EEG study. Hum Brain Mapp. 2019;41:1573–1590. 31816147 10.1002/hbm.24896 PMC7267959

[3] Collerton D , Perry E, McKeith I. Why people see things that are not there: A novel perception and attention deficit model for recurrent complex visual hallucinations. Behav Brain Sci. 2005;28:737–757. 16372931 10.1017/S0140525X05000130

[4] McKeith I . Dementia with Lewy bodies. In: Handbook of clinical neurology. Elsevier; 2007:531–548. 10.1016/S0072-9752(07)84060-7 18808969

[5] Mosimann UP , Collerton D, Dudley R, et al A semi-structured interview to assess visual hallucinations in older people. Int J Geriatr Psychiatry. 2008;23:712–718. 18181237 10.1002/gps.1965

[6] Weil RS , Schrag AE, Warren JD, Crutch SJ, Lees AJ, Morris HR. Visual dysfunction in Parkinson’s disease. Brain. 2016;139:2827–2843. 27412389 10.1093/brain/aww175 PMC5091042

[7] Aarsland D , Ballard C, Larsen JP, McKeith I. A comparative study of psychiatric symptoms in dementia with Lewy bodies and Parkinson’s disease with and without dementia. Int J Geriatr Psychiatry. 2001;16:528–536. 11376470 10.1002/gps.389

[8] Burghaus L , Eggers C, Timmermann L, Fink GR, Diederich NJ. Hallucinations in neurodegenerative diseases. CNS Neurosci Ther. 2012;18:149–159. 21592320 10.1111/j.1755-5949.2011.00247.x PMC6493408

[9] Fénelon G , Mahieux F, Huon R, Ziégler M. Hallucinations in Parkinson’s disease: prevalence, phenomenology and risk factors. Brain. 2000;123:733–745. 10734005 10.1093/brain/123.4.733

[10] Barnes J , David AS. Visual hallucinations in Parkinson’s disease: A review and phenomenological survey. J Neurol Neurosurg Psychiatry. 2001;70:727–733. 11385004 10.1136/jnnp.70.6.727 PMC1737396

[11] Holroyd S , Currie L, Wooten GF. Prospective study of hallucinations and delusions in Parkinson’s disease. J Neurol Neurosurg Psychiatry. 2001;70:734–738. 11385005 10.1136/jnnp.70.6.734 PMC1737419

[12] Tiraboschi P , Salmon DP, Hansen LA, Hofstetter RC, Thal LJ, Corey-Bloom J. What best differentiates Lewy body from Alzheimer’s disease in early-stage dementia? Brain. 2006;129:729–735. 16401618 10.1093/brain/awh725

[13] Toledo JB , Cairns NJ, Da X, et al Clinical and multimodal biomarker correlates of ADNI neuropathological findings. Acta Neuropathol Commun. 2013;1:65. 24252435 10.1186/2051-5960-1-65 PMC3893373

[14] Yoshizawa H , Vonsattel JPG, Honig LS. Early neuropsychological discriminants for Lewy body disease: An autopsy series. J Neurol Neurosurg Psychiatry. 2013;84:1326–1330. 23308020 10.1136/jnnp-2012-304381

[15] Jicha GA , Schmitt FA, Abner E, et al Prodromal clinical manifestations of neuropathologically confirmed Lewy body disease. Neurobiol Aging. 2010;31:1805–1813. 19026468 10.1016/j.neurobiolaging.2008.09.017 PMC2891418

[16] Ibarretxe-Bilbao N , Ramirez-Ruiz B, Junque C, et al Differential progression of brain atrophy in Parkinson’s disease with and without visual hallucinations. J Neurol Neurosurg Psychiatry. 2010;81:650–657. 19965847 10.1136/jnnp.2009.179655

[17] Ballard C , Piggott M, Johnson M, et al Delusions associated with elevated muscarinic binding in dementia with Lewy bodies. Ann Neurol. 2000;48:868–876. 11117543

[18] Diederich NJ , Goetz CG, Stebbins GT. Repeated visual hallucinations in Parkinson’s disease as disturbed external/internal perceptions: Focused review and a new integrative model. Mov Disord. 2005;20:130–140. 15486924 10.1002/mds.20308

[19] Harding AJ , Broe GA, Halliday GM. Visual hallucinations in Lewy body disease relate to Lewy bodies in the temporal lobe. Brain. 2002;125:391–403. 11844739 10.1093/brain/awf033

[20] Onofrj M , Espay AJ, Bonanni L, Delli Pizzi S, Sensi SL. Hallucinations, somatic-functional disorders of PD-DLB as expressions of thalamic dysfunction. Mov Disord. 2019;34:1100–1111. 31307115 10.1002/mds.27781 PMC6707070

[21] Shine JM , Halliday GM, Naismith SL, Lewis SJ. Visual misperceptions and hallucinations in Parkinson’s disease: Dysfunction of attentional control networks? Mov Disord. 2011;26:2154–2159. 21953814 10.1002/mds.23896

[22] Shine JM , Halliday GM, Gilat M, et al The role of dysfunctional attentional control networks in visual misperceptions in Parkinson’s disease. Hum Brain Mapp. 2014;35:2206–2219. 23760982 10.1002/hbm.22321 PMC6869072

[23] Tsukada H , Fujii H, Aihara K, Tsuda I. Computational model of visual hallucination in dementia with Lewy bodies. Neural Networks. 2015;62:73–82. 25282547 10.1016/j.neunet.2014.09.001

[24] Benrimoh D , Parr T, Adams RA, Friston K. Hallucinations both in and out of context: An active inference account. PLoS ONE. 2019;14:e0212379. 31430277 10.1371/journal.pone.0212379 PMC6701798

[25] Pezzoli S , Cagnin A, Antonini A, Venneri A. Frontal and subcortical contribution to visual hallucinations in dementia with Lewy bodies and Parkinson’s disease. Postgrad Med. 2019;131:509–522. 31422718 10.1080/00325481.2019.1656515

[26] Gasca-Salas C , Clavero P, García-García D, Obeso JA, Rodríguez-Oroz MC. Significance of visual hallucinations and cerebral hypometabolism in the risk of dementia in Parkinson’s disease patients with mild cognitive impairment. Hum Brain Mapp. 2016;37:968–977. 26663702 10.1002/hbm.23080 PMC6867477

[27] Pasquier J , Michel BF, Brenot-Rossi I, Hassan-Sebbag N, Sauvan R, Gastaut JL. Value of (99 m)Tc-ECD SPET for the diagnosis of dementia with Lewy bodies. Eur J Nucl Med Mol Imaging. 2002;29:1342–1348. 12271417 10.1007/s00259-002-0919-x

[28] Taylor J-P , Firbank MJ, He J, et al Visual cortex in dementia with Lewy bodies: Magnetic resonance imaging study. Br J Psychiatry. 2012;200:491–498. 22500014 10.1192/bjp.bp.111.099432 PMC3365275

[29] Firbank MJ , Parikh J, Murphy N, et al Reduced occipital GABA in Parkinson disease with visual hallucinations. Neurology. 2018;91:e675–e685. 30021920 10.1212/WNL.0000000000006007 PMC6105043

[30] Barnes J , Boubert L, Harris J, Lee A, David AS. Reality monitoring and visual hallucinations in Parkinson’s disease. Neuropsychologia. 2003;41:565–574. 12559149 10.1016/s0028-3932(02)00182-3

[31] Franciotti R , Delli Pizzi S, Perfetti B, et al Default mode network links to visual hallucinations: A comparison between Parkinson’s disease and multiple system atrophy. Mov Disord. 2015;30:1237–1247. 26094856 10.1002/mds.26285

[32] Yao N , Shek-Kwan Chang R, Cheung C, et al The default mode network is disrupted in Parkinson’s disease with visual hallucinations. Hum Brain Mapp. 2014;35:5658–5666. 24985056 10.1002/hbm.22577 PMC4657500

[33] Hepp DH , Foncke EMJ, Dubbelink KTEO, Berg WDJVD, Berendse HW, Schoonheim MM. Loss of functional connectivity in patients with Parkinson disease and visual hallucinations. Radiology. 2017;285:896–903. 28952907 10.1148/radiol.2017170438

[34] Ffytche DH . The hodology of hallucinations. Cortex. 2008;44:1067–1083. 18586234 10.1016/j.cortex.2008.04.005

[35] de Haan W , Pijnenburg YA, Strijers RL, et al Functional neural network analysis in frontotemporal dementia and Alzheimer’s disease using EEG and graph theory. BMC Neuroscience. 2009;10:101. 19698093 10.1186/1471-2202-10-101 PMC2736175

[36] Acharya UR , Sree SV, Swapna G, Martis RJ, Suri JS. Automated EEG analysis of epilepsy: A review. Knowl Based Syst. 2013;45:147–165.

[37] Furlong S , Cohen JR, Hopfinger J, Snyder J, Robertson MM, Sheridan MA. Resting-state EEG connectivity in young children with ADHD. J Clin Child Adolesc Psychol. 2021;50:1–17. 32809852 10.1080/15374416.2020.1796680 PMC7889746

[38] Kurita A , Murakami M, Takagi S, Matsushima M, Suzuki M. Visual hallucinations and altered visual information processing in Parkinson disease and dementia with Lewy bodies. Mov Disord. 2010;25:167–71. 20063433 10.1002/mds.22919

[39] Kurita A , Nakamura M, Suzuki M, Mochio S, Inoue K. Visual and auditory event-related potential comparisons between Parkinson’s disease with dementia and Alzheimer’s disease. Int Congr Series. 2005;1278:57–60.

[40] Matsui H , Udaka F, Tamura A, et al The relation between visual hallucinations and visual evoked potential in Parkinson disease. Clin Neuropharmacol. 2005;28:79–82. 15795550 10.1097/01.wnf.0000157066.50948.65

[41] Chang Y-P , Yang Y-H, Lai C-L, Liou L-M. Event-related potentials in Parkinson’s disease patients with visual hallucination. Parkinsons Dis. 2016;2016:1863508. 28053801 10.1155/2016/1863508 PMC5178355

[42] Dauwan M , Linszen MMJ, Lemstra AW, Scheltens P, Stam CJ, Sommer IE. EEG-based neurophysiological indicators of hallucinations in Alzheimer’s disease: Comparison with dementia with Lewy bodies. Neurobiol Aging. 2018;67:75–83. 29653315 10.1016/j.neurobiolaging.2018.03.013

[43] Dauwan M , Hoff JI, Vriens EM, Hillebrand A, Stam CJ, Sommer IE. Aberrant resting-state oscillatory brain activity in Parkinson’s disease patients with visual hallucinations: An MEG source-space study. NeuroImage Clin. 2019;22:101752. 30897434 10.1016/j.nicl.2019.101752 PMC6425119

[44] Babiloni C , Pascarelli MT, Lizio R, et al Abnormal cortical neural synchronization mechanisms in quiet wakefulness are related to motor deficits, cognitive symptoms, and visual hallucinations in Parkinson’s disease patients: An electroencephalographic study. Neurobiol Aging. 2020;91:88–111. 32234263 10.1016/j.neurobiolaging.2020.02.029

[45] Schumacher J , Thomas AJ, Peraza LR, et al EEG alpha reactivity and cholinergic system integrity in Lewy body dementia and Alzheimer’s disease. Alzheimers Res Ther. 2020;12:46. 32321573 10.1186/s13195-020-00613-6 PMC7178985

[46] Kai T , Asai Y, Sakuma K, Koeda T, Nakashima K. Quantitative electroencephalogram analysis in dementia with Lewy bodies and Alzheimer’s disease. J Neurol Sci. 2005;237:89–95. 16019033 10.1016/j.jns.2005.05.017

[47] Agnoli A , Martucci N, Manna V, Conti L, Fioravanti M. Effect of cholinergic and anticholinergic drugs on short-term memory in Alzheimer’s dementia: A neuropsychological and computerized electroencephalographic study. Clin Neuropharmacol. 1983;6:311–323. 6661730 10.1097/00002826-198312000-00005

[48] Balkan S , Yaras N, Mihci E, Dora B, Agar A, Yargiçoglu P. Effect of donepezil on EEG spectral analysis in Alzheimer’s disease. Acta Neurol Belg. 2003;103:164–169. 14626697

[49] Perry EK , Perry RH. Acetylcholine and hallucinations: Disease-related compared to drug-induced alterations in human consciousness. Brain Cogn. 1995;28:240–258. 8546852 10.1006/brcg.1995.1255

[50] Hepp DH , Foncke EMJ, Berendse HW, et al Damaged fiber tracts of the nucleus basalis of Meynert in Parkinson’s disease patients with visual hallucinations. Sci Rep. 2017;7:10112–10112. 28860465 10.1038/s41598-017-10146-y PMC5579278

[51] Sakai K , Ikeda T, Ishida C, Komai K, Yamada M. Delusions and visual hallucinations in a patient with Parkinson’s disease with dementia showing pronounced Lewy body pathology in the nucleus basalis of Meynert. Neuropathology. 2019;39:319–323. 31243794 10.1111/neup.12581

[52] Alexander AL , Lee JE, Lazar M, Field AS. Diffusion tensor imaging of the brain. Neurotherapeutics. 2007;4:316–329. 17599699 10.1016/j.nurt.2007.05.011 PMC2041910

[53] Johansen-Berg H , Behrens TE. Diffusion MRI: From quantitative measurement to in vivo neuroanatomy: Academic Press; 2013.

[54] Chu CJ , Tanaka N, Diaz J, et al EEG functional connectivity is partially predicted by underlying white matter connectivity. NeuroImage. 2015;108:23–33. 25534110 10.1016/j.neuroimage.2014.12.033 PMC4323839

[55] Duru AD , Duru DG, Yumerhodzha S, Bebek N. Analysis of correlation between white matter changes and functional responses in thalamic stroke: A DTI & EEG study. Brain Imaging Behav. 2016;10:424–436. 25957181 10.1007/s11682-015-9397-1

[56] Nunez PL , Srinivasan R, Fields RD. EEG functional connectivity, axon delays and white matter disease. Clin Neurophysiol. 2015;126:110–120. 24815984 10.1016/j.clinph.2014.04.003 PMC5018992

[57] Pizzi S D , Maruotti V, Taylor J-P, et al Relevance of subcortical visual pathways disruption to visual symptoms in dementia with Lewy bodies. Cortex. 2014;59:12–21. 25113955 10.1016/j.cortex.2014.07.003

[58] Zarkali A , McColgan P, Ryten M, et al Differences in network controllability and regional gene expression underlie hallucinations in Parkinson’s disease. Brain. 2020;143:3435–3448. 33118028 10.1093/brain/awaa270 PMC7719028

[59] Zarkali A , McColgan P, Leyland L-A, Lees AJ, Rees G, Weil RS. Fiber-specific white matter reductions in Parkinson hallucinations and visual dysfunction. Neurology. 2020;94:e1525–e1538. 32094242 10.1212/WNL.0000000000009014 PMC7251523

[60] Klimesch W , Doppelmayr M, Russegger H, Pachinger T, Schwaiger J. Induced alpha band power changes in the human EEG and attention. Neurosci Lett. 1998;244:73–76. 9572588 10.1016/s0304-3940(98)00122-0

[61] Mulholland T , Runnals S. Increased occurrence of EEG alpha during increased attention. J Psychol. 1962;54:317–330.

[62] Benedek M , Schickel RJ, Jauk E, Fink A, Neubauer AC. Alpha power increases in right parietal cortex reflects focused internal attention. Neuropsychologia. 2014;56:393–400. 24561034 10.1016/j.neuropsychologia.2014.02.010 PMC3989020

[63] Wan L , Huang H, Schwab N, et al From eyes-closed to eyes-open: role of cholinergic projections in EC-to-EO alpha reactivity revealed by combining EEG and MRI. Hum Brain Mapp. 2019;40:566–577. 30251753 10.1002/hbm.24395 PMC6338213

[64] Chapman RM , Armington JC, Bragdon HR. A quantitative survey of kappa and alpha EEG activity. Electroencephalogr Clin Neurophysiol. 1962;14:858–868. 14020161 10.1016/0013-4694(62)90136-0

[65] Jensen O , Mazaheri A. Shaping functional architecture by oscillatory alpha activity: Gating by inhibition. Front Hum Neurosci. 2010;4:186. 21119777 10.3389/fnhum.2010.00186 PMC2990626

[66] Briel RC , McKeith IG, Barker WA, et al EEG findings in dementia with Lewy bodies and Alzheimer’s disease. J Neurol Neurosurg Psychiatry. 1999;66:401–403. 10084544 10.1136/jnnp.66.3.401 PMC1736269

[67] Peraza LR , Cromarty R, Kobeleva X, et al Electroencephalographic derived network differences in Lewy body dementia compared to Alzheimer’s disease patients. Sci Rep. 2018;8:4637. 29545639 10.1038/s41598-018-22984-5 PMC5854590

[68] Lopes da Silva FH , van Lierop THMT, Schrijer CF, Storm van Leeuwen W. Organization of thalamic and cortical alpha rhythms: Spectra and coherences. Electroencephalogr Clin Neurophysiol. 1973;35:627–639. 4128158 10.1016/0013-4694(73)90216-2

[69] Schürmann M , Demiralp T, Başar E, Başar Eroglu C. Electroencephalogram alpha (8–15 Hz) responses to visual stimuli in cat cortex, thalamus, and hippocampus: A distributed alpha network? Neurosci Lett. 2000;292:175–178. 11018305 10.1016/s0304-3940(00)01456-7

[70] Roberts J , Robinson P. Modeling distributed axonal delays in mean-field brain dynamics. Phys Rev E. 2008;78:051901. 10.1103/PhysRevE.78.051901 19113149

[71] Robinson P , Rennie C, Wright J, Bahramali H, Gordon E, Rowe D. Prediction of electroencephalographic spectra from neurophysiology. Phys Rev E. 2001;63:021903. 10.1103/PhysRevE.63.021903 11308514

[72] Delli Pizzi S , Franciotti R, Taylor JP, et al Thalamic involvement in fluctuating cognition in dementia with Lewy bodies: Magnetic resonance evidences. Cereb Cortex. 2015;25:3682–3689. 25260701 10.1093/cercor/bhu220 PMC4585510

[73] Schumacher J , Peraza LR, Firbank M, et al Dysfunctional brain dynamics and their origin in Lewy body dementia. Brain. 2019;142:1767–1782. 30938426 10.1093/brain/awz069 PMC6536851

[74] Bar M . A cortical mechanism for triggering top-down facilitation in visual object recognition. J Cogn Neurosci. 2003;15:600–609. 12803970 10.1162/089892903321662976

[75] Chaumon M , Kveraga K, Barrett LF, Bar M. Visual predictions in the orbitofrontal cortex rely on associative content. Cereb Cortex. 2014;24:2899–2907. 23771980 10.1093/cercor/bht146 PMC4193460

[76] Vossel S , Geng JJ, Fink GR. Dorsal and ventral attention systems: Distinct neural circuits but collaborative roles. Neuroscientist. 2014;20:150–159. 23835449 10.1177/1073858413494269 PMC4107817

[77] McKeith IG , Boeve BF, Dickson DW, et al Diagnosis and management of dementia with Lewy bodies: Fourth consensus report of the DLB Consortium. Neurology. 2017;89:88–100. 28592453 10.1212/WNL.0000000000004058 PMC5496518

[78] McKeith IG , Dickson DW, Lowe J, et al Diagnosis and management of dementia with Lewy bodies: Third report of the DLB Consortium. Neurology. 2005;65:1863–1872. 16237129 10.1212/01.wnl.0000187889.17253.b1

[79] Emre M , Aarsland D, Brown R, et al Clinical diagnostic criteria for dementia associated with Parkinson’s disease. Mov Disord. 2007;22:1689–1707. 17542011 10.1002/mds.21507

[80] Folstein MF , Folstein SE, McHugh PR. “Mini-mental state”. A practical method for grading the cognitive state of patients for the clinician. J Psychiatr Res. 1975;12:189–198. 1202204 10.1016/0022-3956(75)90026-6

[81] Tomlinson CL , Stowe R, Patel S, Rick C, Gray R, Clarke CE. Systematic review of levodopa dose equivalency reporting in Parkinson’s disease. Mov Disord. 2010;25:2649–2653. 21069833 10.1002/mds.23429

[82] Oostenveld R , Praamstra P. The five percent electrode system for high-resolution EEG and ERP measurements. Clin Neurophysiol. 2001;112:713–719. 11275545 10.1016/s1388-2457(00)00527-7

[83] Delorme A , Makeig S. EEGLAB: An open source toolbox for analysis of single-trial EEG dynamics including independent component analysis. J Neurosci Methods. 2004;134:9–21. 15102499 10.1016/j.jneumeth.2003.10.009

[84] Bell AJ , Sejnowski TJ. An information-maximization approach to blind separation and blind deconvolution. Neural Comput. 1995;7:1129–1159. 7584893 10.1162/neco.1995.7.6.1129

[85] McMenamin BW , Shackman AJ, Maxwell JS, et al Validation of ICA-based myogenic artifact correction for scalp and source-localized EEG. NeuroImage. 2010;49:2416–2432. 19833218 10.1016/j.neuroimage.2009.10.010 PMC2818255

[86] Delorme A , Sejnowski T, Makeig S. Enhanced detection of artifacts in EEG data using higher-order statistics and independent component analysis. NeuroImage. 2007;34:1443–1449. 17188898 10.1016/j.neuroimage.2006.11.004 PMC2895624

[87] Peraza LR , Kaiser M, Firbank M, et al fMRI resting state networks and their association with cognitive fluctuations in dementia with Lewy bodies. NeuroImage Clin. 2014;4:558–565. 24818081 10.1016/j.nicl.2014.03.013 PMC3984441

[88] Dale AM , Fischl B, Sereno MI. Cortical surface-based analysis: I. segmentation and surface reconstruction. NeuroImage. 1999;9:179–194. 9931268 10.1006/nimg.1998.0395

[89] Fischl B , Dale AM. Measuring the thickness of the human cerebral cortex from magnetic resonance images. Proc Natl Acad Sci USA. 2000;97:11050–11055. 10984517 10.1073/pnas.200033797 PMC27146

[90] Destrieux C , Fischl B, Dale A, Halgren E. Automatic parcellation of human cortical gyri and sulci using standard anatomical nomenclature. NeuroImage. 2010;53:1–15. 20547229 10.1016/j.neuroimage.2010.06.010 PMC2937159

[91] Blanc F , Colloby SJ, Philippi N, et al Cortical thickness in dementia with Lewy bodies and Alzheimer’s disease: A comparison of prodromal and dementia stages. PLoS ONE. 2015;10:e0127396. 26061655 10.1371/journal.pone.0127396 PMC4489516

[92] Colloby SJ , Firbank MJ, Vasudev A, Parry SW, Thomas AJ, O’Brien JT. Cortical thickness and VBM-DARTEL in late-life depression. J Affect Disord. 2011;133:158–164. 21550668 10.1016/j.jad.2011.04.010

[93] Firbank MJ , Blamire AM, Krishnan MS, et al Diffusion tensor imaging in dementia with Lewy bodies and Alzheimer’s disease. Psychiatry Res. 2007;155:135–145. 17408930 10.1016/j.pscychresns.2007.01.001

[94] Andersson JLR , Sotiropoulos SN. An integrated approach to correction for off-resonance effects and subject movement in diffusion MR imaging. NeuroImage. 2016;125:1063–1078. 26481672 10.1016/j.neuroimage.2015.10.019 PMC4692656

[95] Andersson JLR , Graham MS, Zsoldos E, Sotiropoulos SN. Incorporating outlier detection and replacement into a non-parametric framework for movement and distortion correction of diffusion MR images. NeuroImage. 2016;141:556–572. 27393418 10.1016/j.neuroimage.2016.06.058

[96] Pascual-Marqui RD . Standardized low-resolution brain electromagnetic tomography (sLORETA): Technical details. Methods Find Exp Clin Pharmacol. 2002;24:5–12. 12575463

[97] Tadel F , Baillet S, Mosher JC, Pantazis D, Leahy RM. Brainstorm: A user-friendly application for MEG/EEG analysis. Comput Intell Neurosci. 2011;2011:879716. 21584256 10.1155/2011/879716 PMC3090754

[98] Grech R , Cassar T, Muscat J, et al Review on solving the inverse problem in EEG source analysis. J Neuroeng Rehabil. 2008;5:25. 18990257 10.1186/1743-0003-5-25 PMC2605581

[99] Hincapié A-S , Kujala J, Mattout J, et al The impact of MEG source reconstruction method on source-space connectivity estimation: A comparison between minimum-norm solution and beamforming. NeuroImage. 2017;156:29–42. 28479475 10.1016/j.neuroimage.2017.04.038

[100] Stropahl M , Bauer A-KR, Debener S, Bleichner MG. Source-modeling auditory processes of EEG data using EEGLAB and brainstorm. protocols. Front Neurosci. 2018;12:309. 29867321 10.3389/fnins.2018.00309 PMC5952032

[101] Gramfort A , Papadopoulo T, Olivi E, Clerc M. OpenMEEG: Opensource software for quasistatic bioelectromagnetics. Biomed Eng Online. 2010;9:45. 20819204 10.1186/1475-925X-9-45 PMC2949879

[102] Kybic J , Clerc M, Abboud T, Faugeras O, Keriven R, Papadopoulo T. A common formalism for the Integral formulations of the forward EEG problem. IEEE Trans Med Imaging. 2005;24:12–28. 15638183 10.1109/tmi.2004.837363

[103] Vinck M , Oostenveld R, van Wingerden M, Battaglia F, Pennartz CM. An improved index of phase-synchronization for electrophysiological data in the presence of volume-conduction, noise and sample-size bias. NeuroImage. 2011;55:1548–1565. 21276857 10.1016/j.neuroimage.2011.01.055

[104] Peraza LR , Asghar AUR, Green G, Halliday DM. Volume conduction effects in brain network inference from electroencephalographic recordings using phase lag index. J Neurosci Methods. 2012;207:189–199. 22546477 10.1016/j.jneumeth.2012.04.007

[105] Oostenveld R , Fries P, Maris E, Schoffelen JM. FieldTrip: Open source software for advanced analysis of MEG, EEG, and invasive electrophysiological data. Comput Intell Neurosci. 2011;2011:156869. 21253357 10.1155/2011/156869 PMC3021840

[106] Rubinov M , Sporns O. Complex network measures of brain connectivity: Uses and interpretations. NeuroImage. 2010;52:1059–1069. 19819337 10.1016/j.neuroimage.2009.10.003

[107] Newman ME . Fast algorithm for detecting community structure in networks. Phys Rev E. 2004;69:066133. 10.1103/PhysRevE.69.066133 15244693

[108] Opsahl T , Agneessens F, Skvoretz J. Node centrality in weighted networks: generalizing degree and shortest paths. Soc Netw. 2010;32:245–251.

[109] Zalesky A , Fornito A, Bullmore ET. Network-based statistic: Identifying differences in brain networks. NeuroImage. 2010;53:1197–207. 20600983 10.1016/j.neuroimage.2010.06.041

[110] Xia M , Wang J, He Y. BrainNet viewer: A network visualization tool for human brain connectomics. PLoS ONE. 2013;8:e68910. 23861951 10.1371/journal.pone.0068910 PMC3701683

[111] Onnela J-P , Saramäki J, Kertész J, Kaski K. Intensity and coherence of motifs in weighted complex networks. Phys Rev E. 2005;71:065103. 10.1103/PhysRevE.71.065103 16089800

[112] Lancichinetti A , Fortunato S. Consensus clustering in complex networks. Sci Rep. 2012;2:336. 22468223 10.1038/srep00336 PMC3313482

[113] Eickhoff SB , Stephan KE, Mohlberg H, et al A new SPM toolbox for combining probabilistic cytoarchitectonic maps and functional imaging data. NeuroImage. 2005;25:1325–1335. 15850749 10.1016/j.neuroimage.2004.12.034

[114] Behrens TEJ , Johansen-Berg H, Woolrich MW, et al Non-invasive mapping of connections between human thalamus and cortex using diffusion imaging. Nat Neurosci. 2003;6:750–757. 12808459 10.1038/nn1075

[115] Jenkinson M , Beckmann CF, Behrens TE, Woolrich MW, Smith SM. FSL. NeuroImage. 2012;62:782–790. 21979382 10.1016/j.neuroimage.2011.09.015

[116] Avants BB , Tustison N, Song G. Advanced normalization tools (ANTS). Insight J. 2009;2:1–35.

[117] Jenkinson M , Bannister P, Brady M, Smith S. Improved optimization for the robust and accurate linear registration and motion correction of brain images. NeuroImage. 2002;17:825–841. 12377157 10.1016/s1053-8119(02)91132-8

[118] Behrens TE , Woolrich MW, Jenkinson M, et al Characterization and propagation of uncertainty in diffusion-weighted MR imaging. Magn Reson Med. 2003;50:1077–1088. 14587019 10.1002/mrm.10609

[119] Behrens TE , Berg HJ, Jbabdi S, Rushworth MF, Woolrich MW. Probabilistic diffusion tractography with multiple fibre orientations: What can we gain? NeuroImage. 2007;34:144–155. 17070705 10.1016/j.neuroimage.2006.09.018 PMC7116582

[120] Cabral J , Hugues E, Sporns O, Deco G. Role of local network oscillations in resting-state functional connectivity. NeuroImage. 2011;57:130–139. 21511044 10.1016/j.neuroimage.2011.04.010

[121] Perneczky R , Drzezga A, Boecker H, Förstl H, Kurz A, Häussermann P. Cerebral metabolic dysfunction in patients with dementia with Lewy bodies and visual hallucinations. Dementia Geriatr Cognit Disord. 2008;25:531–538. 10.1159/000132084 18477846

[122] Luders HO . Textbook of epilepsy surgery: CRC Press; 2008.

[123] Livingstone MS , Hubel DH. Psychophysical evidence for separate channels for the perception of form, color, movement, and depth. J Neurosci. 1987;7:3416–3468. 3316524 10.1523/JNEUROSCI.07-11-03416.1987 PMC6569044

[124] Morris JS , Öhman A, Dolan RJ. A subcortical pathway to the right amygdala mediating “unseen” fear. Proc Natl Acad Sci USA. 1999;96:1680–1685. 9990084 10.1073/pnas.96.4.1680 PMC15559

[125] Bar M , Kassam KS, Ghuman AS, et al Top-down facilitation of visual recognition. Proc Natl Acad Sci USA. 2006;103:449–454. 16407167 10.1073/pnas.0507062103 PMC1326160

[126] Kveraga K , Ghuman AS, Bar M. Top-down predictions in the cognitive brain. Brain Cogn. 2007;65:145–168. 17923222 10.1016/j.bandc.2007.06.007 PMC2099308

[127] Lundstrom BN , Petersson KM, Andersson J, Johansson M, Fransson P, Ingvar M. Isolating the retrieval of imagined pictures during episodic memory: Activation of the left precuneus and left prefrontal cortex. NeuroImage. 2003;20:1934–1943. 14683699 10.1016/j.neuroimage.2003.07.017

[128] Nolde SF , Johnson MK, Raye CL. The role of prefrontal cortex during tests of episodic memory. Trends Cogn Sci. 1998;2:399–406. 21227255 10.1016/s1364-6613(98)01233-9

[129] Fletcher PC , Henson RNA. Frontal lobes and human memory: Insights from functional neuroimaging. Brain. 2001;124:849–881. 11335690 10.1093/brain/124.5.849

[130] Summerfield C , Egner T, Greene M, Koechlin E, Mangels J, Hirsch J. Predictive codes for forthcoming perception in the frontal cortex. Science. 2006;314:1311–1314. 17124325 10.1126/science.1132028

[131] Lang X , Wang L, Zhuo C-J, Jia F, Wang L-N, Wang C-L. Reduction of interhemispheric functional connectivity in sensorimotor and visual information processing pathways in schizophrenia. Chin Med J. 2016;129:2422–2426. 27748333 10.4103/0366-6999.191758 PMC5072253

[132] Chang X , Xi YB, Cui LB, et al Distinct inter-hemispheric dysconnectivity in schizophrenia patients with and without auditory verbal hallucinations. Sci Rep. 2015;5:11218. 26053998 10.1038/srep11218 PMC4459220

[133] Wigand M , Kubicki M, von Hohenberg CC, et al Auditory verbal hallucinations and the interhemispheric auditory pathway in chronic schizophrenia. World J Biol Psychiatry. 2015;16:31–44. 25224883 10.3109/15622975.2014.948063 PMC4698973

[134] Mima T , Oluwatimilehin T, Hiraoka T, Hallett M. Transient interhemispheric neuronal synchrony correlates with object recognition. J Neurosci. 2001;21:3942–3948. 11356882 10.1523/JNEUROSCI.21-11-03942.2001 PMC6762719

[135] Raichle ME . The brain’s default mode network. Ann Rev Neurosci. 2015;38:433–447. 25938726 10.1146/annurev-neuro-071013-014030

[136] Jimenez AM , Lee J, Wynn JK, et al Abnormal ventral and dorsal attention network activity during single and dual target detection in schizophrenia. Front Psychol. 2016;7:323. 27014135 10.3389/fpsyg.2016.00323 PMC4781842

[137] Krall SC , Rottschy C, Oberwelland E, et al The role of the right temporoparietal junction in attention and social interaction as revealed by ALE meta-analysis. Brain Struct Funct. 2015;220:587–604. 24915964 10.1007/s00429-014-0803-z PMC4791048

[138] Blanc F , Noblet V, Philippi N, et al Right anterior insula: Core region of hallucinations in cognitive neurodegenerative diseases. PLoS ONE. 2014;9:e114774. 25479196 10.1371/journal.pone.0114774 PMC4257732

[139] Treisman AM , Kanwisher NG. Perceiving visually presented objects: Recognition, awareness, and modularity. Curr Opin Neurobiol. 1998;8:218–226. 9635205 10.1016/s0959-4388(98)80143-8

[140] Zeki S , Bartels A. The autonomy of the visual systems and the modularity of conscious vision. Philos Trans R Soc Lond B Biol Sci. 1998;353:1911–1914. 9854263 10.1098/rstb.1998.0343 PMC1692424

[141] Borowsky R , Loehr J, Kelland Friesen C, Kraushaar G, Kingstone A, Sarty G. Modularity and intersection of “what”, “where” and “how” processing of visual stimuli: a new method of FMRI localization. Brain Topogr. 2005;18:67–75. 16341575 10.1007/s10548-005-0276-8

[142] O’Callaghan C , Kveraga K, Shine JM, Adams RB, Bar M. Predictions penetrate perception: Converging insights from brain, behaviour and disorder. Conscious Cogn. 2017;47:63–74. 27222169 10.1016/j.concog.2016.05.003 PMC5764074

[143] Gamond L , George N, Lemaréchal J-D, Hugueville L, Adam C, Tallon-Baudry C. Early influence of prior experience on face perception. NeuroImage. 2011;54:1415–1426. 20832479 10.1016/j.neuroimage.2010.08.081

[144] Chaumon M , Hasboun D, Baulac M, Adam C, Tallon-Baudry C. Unconscious contextual memory affects early responses in the anterior temporal lobe. Brain Res. 2009;1285:77–87. 19505440 10.1016/j.brainres.2009.05.087

[145] Zhang R , Shao R, Xu G, et al Aberrant brain structural–functional connectivity coupling in euthymic bipolar disorder. Hum Brain Mapp. 2019;40:3452–3463. 31282606 10.1002/hbm.24608 PMC6865442

[146] Li K , Liu L, Yin Q, et al Abnormal rich club organization and impaired correlation between structural and functional connectivity in migraine sufferers. Brain Imaging Behav. 2017;11:526–540. 26922054 10.1007/s11682-016-9533-6

[147] Guye M , Parker GJM, Symms M, et al Combined functional MRI and tractography to demonstrate the connectivity of the human primary motor cortex in vivo. NeuroImage. 2003;19:1349–1360. 12948693 10.1016/s1053-8119(03)00165-4

[148] Greicius MD , Supekar K, Menon V, Dougherty RF. Resting-state functional connectivity reflects structural connectivity in the default mode network. Cereb Cortex. 2008;19:72–78. 18403396 10.1093/cercor/bhn059 PMC2605172

[149] Hindriks R , Woolrich M, Luckhoo H, et al Role of white-matter pathways in coordinating alpha oscillations in resting visual cortex. NeuroImage. 2015;106:328–339. 25449741 10.1016/j.neuroimage.2014.10.057

[150] Perry EK , Marshall E, Kerwin J, et al Evidence of a monoaminergic-cholinergic imbalance related to visual hallucinations in Lewy body dementia. J Neurochem. 1990;55:1454–1456. 1697897 10.1111/j.1471-4159.1990.tb03162.x

[151] Zarkali A , McColgan P, Leyland L-A, Lees AJ, Weil RS. Visual dysfunction predicts cognitive impairment and white matter degeneration in Parkinson’s disease. Mov Disord. 2021;36:1191–1202. 33421201 10.1002/mds.28477 PMC8248368

[152] Arevalo-Rodriguez I , Smailagic N, Roqué-Figuls M, et al Mini-Mental State Examination (MMSE) for the early detection of dementia in people with mild cognitive impairment (MCI). Cochrane Database Syst Rev. 2021;7:CD010783. 34313331 10.1002/14651858.CD010783.pub3 PMC8406467

[153] Archibald NK , Clarke MP, Mosimann UP, Burn DJ. Visual symptoms in Parkinson’s disease and Parkinson’s disease dementia. Mov Disord. 2011;26:2387–2395. 21953737 10.1002/mds.23891

[154] Pizzi S D , Franciotti R, Tartaro A, et al Structural alteration of the dorsal visual network in DLB patients with visual hallucinations: A cortical thickness MRI study. PLoS ONE. 2014;9:e86624. 24466177 10.1371/journal.pone.0086624 PMC3900597

[155] Benarroch EE . Pedunculopontine nucleus: functional organization and clinical implications. Neurology. 2013;80:1148–1155. 23509047 10.1212/WNL.0b013e3182886a76

[156] Satoh K , Fibiger HC. Cholinergic neurons of the laterodorsal tegmental nucleus: efferent and afferent connections. J Comp Neurol. 1986;253:277–302. 2432101 10.1002/cne.902530302

