# Supplementary material for: Functional and structural brain network correlates of visual hallucinations in Lewy body dementia
Source: Brain. 2022 Mar 9;145(6):2190–205. doi: 10.1093/brain/awac094 (PMC9246710; doi:10.1093/brain/awac094)
Supplement: awac094_Supplementary_Data [file awac094_supplementary_data.pdf]

Supplementary material

Functional and Structural Brain Network Correlates of Visual Hallucinations in Lewy Body Dementia

Authors:

Ramtin Mehraram, Luis R. Peraza, Nicholas R. E. Murphy, Ruth A. Cromarty, Sara Graziadio, John T. O'Brien, Alison Killen, Sean J. Colloby, Michael Firbank, Li Su, Daniel Collerton, John-Paul Taylor and Marcus Kaiser.

Contents

1. Validation of the source localization pipeline
2. Age as a nuisance covariate in the Network Based Statistics
3. Validation of MMSE as cognition-related score
4. Potential effect of cholinergic medication on the functional connectivity analysis
5. DLB and PDD groups
6. EEG network differences: theta and beta frequency band
7. Alternative measures of structural connectivity
8. EEG network coordinates

1. Validation of the source localization pipeline

To validate the implemented cortical source estimation, we used the EEG data collected for a different study involving a motor task paradigm, where participants were asked to maintain isometric contraction by opposition of thumb and index.[1] We randomly chose four subjects, pre-processed the respective EEG task data, estimated the cortical sources, and generated the power spectrum topographies. For the source localization pipeline being deemed correct, we expected a prominent power activation across time points within the β-band over sensory-motor areas.[2-5] This was in fact the case, as shown in Figure S1.

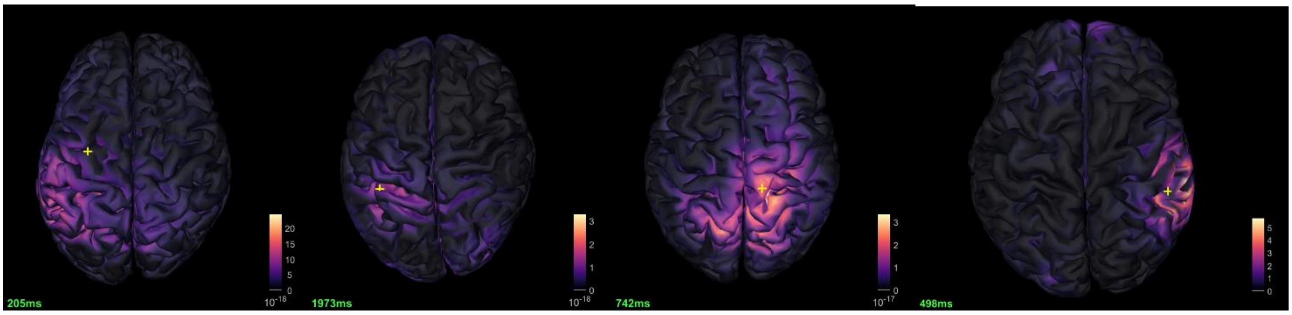

Figure S1 – Topographies of power spectra during a motor task. Topographies were obtained on four randomly chosen healthy control subjects; they all show a prominent activation over the sensorimotor areas.

2. Age as a nuisance covariate in the Network Based Statistics

To address the potential concern on the possible effect of age on the statistics, we also performed the NBS by including age as nuisance covariate and tested the difference in EEG network patterns between groups. As expected, we found a network component which largely resembled the one we reported in the main text (14 edges, 15 nodes, P = 0.033, Figure SII), confirming the robustness of our results.

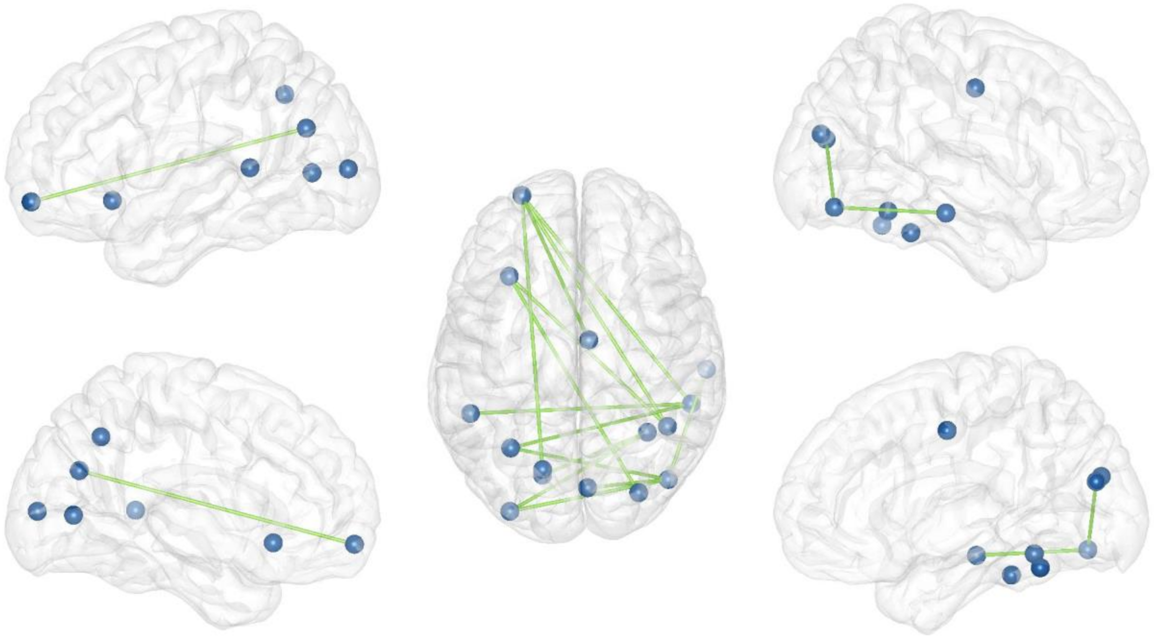

Figure SI1 – Results of the NBS with inclusion of age as a nuisance covariate. The obtained connectome resembles the results reported in the main text.

3. Validation of MMSE as cognition-related score

A potential limitation of our analysis lies in the choice of the MMSE as a cognition-related behavioural score. This score reportedly shows low sensitivity in characterizing the cognitive phenotype of patient groups, and may poorly represent executive function. To address any potential concern, we additionally tested whether any significant difference in the CAMCOG subdomain scores between groups exists. We first compared the CAMCOG total score, and no significant result emerged (P = 0.6442). By including the CAMCOG total score as a nuisance covariate in the NBS, we obtained a differential subnetwork which resembled the one we reported in the main text (P = 0.027, 17 edges, 18 nodes). By testing the individual subdomains, we found a significant difference only for the orientation score (P = 0.033), which was higher for the NVH group. With the inclusion of this score as a covariate in the NBS we still found a resemblant significant component, although with a weaker effect and only by choosing a more permissive primary threshold (t_{th} = 13, P = 0.046). Although not significantly different between groups, we also performed the NBS by including the CAMCOG visuo-perceptual score, as we believed this variable to be pertinent for the purpose of our research. As a result, we found the same network component as the one we reported in the main article. The outcome of this additional analysis confirms the robustness of our results. The detected components are shown in Figure SIII.

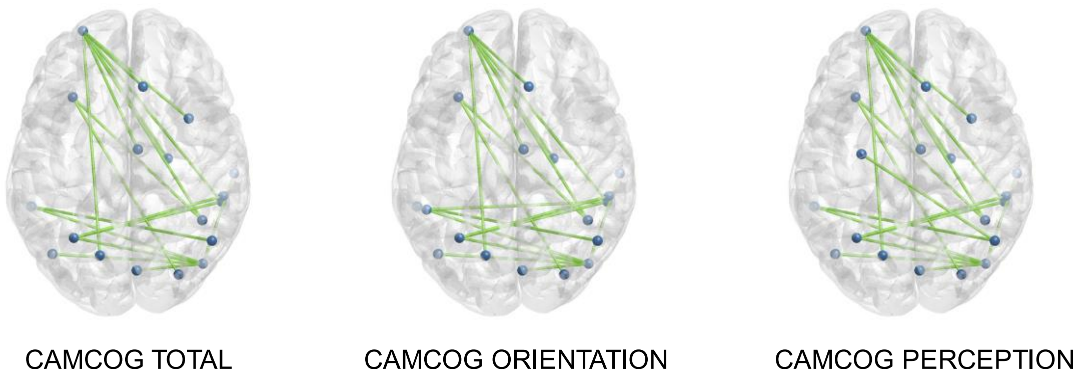

Figure SIII – Results of the NBS with inclusion of CAMCOG scores as a nuisance covariate. The obtained connectomes resemble the results reported in the main text.

4. Potential effect of cholinergic medication on the functional connectivity analysis

To address the potential concern on the effect of cholinergic medication on the functional connectivity analysis, we additionally performed the NBS analysis also including the medication state as a

nuisance covariate. Although we obtained a significant network component, this shows a reduced significance and number of connections compared to the one we reported in the main analysis (P = 0.045, 11 edges, 12 nodes, Figure SIV). This outcome might be due to the low statistical power associated with the imbalance between participants' medication states, as majority of participants were taking cholinesterase inhibitors. Future studies with larger cohorts should better address the effect of this type of medication on functional connectivity alterations associated with LBD-VH as detected with EEG.

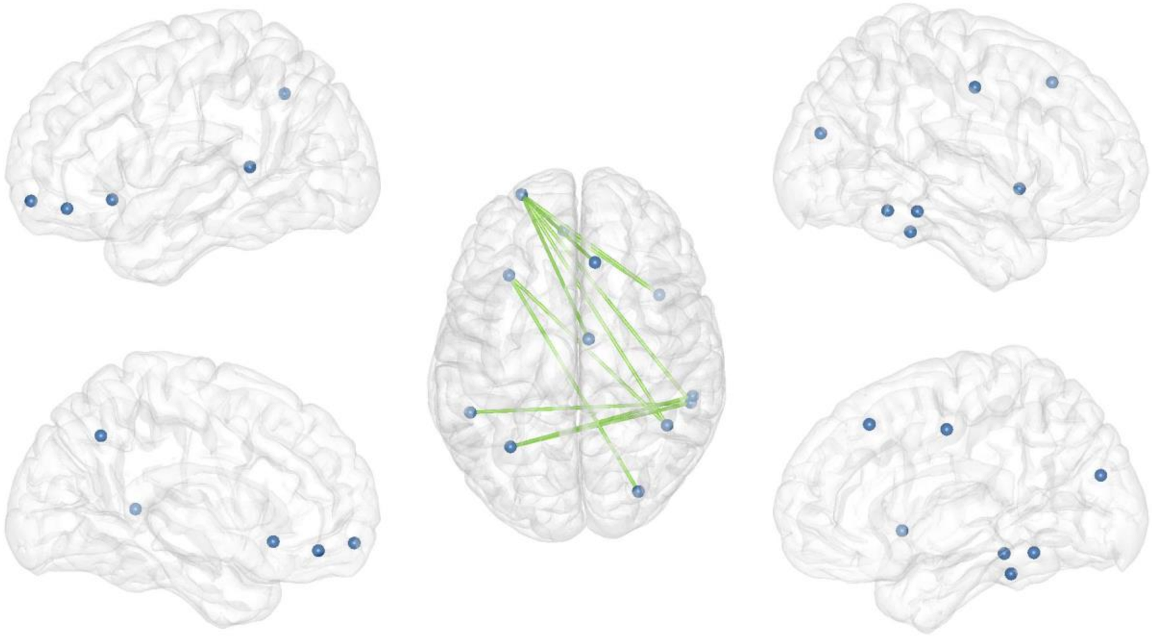

Figure SIV – Results of the NBS with inclusion of medication state as a nuisance covariate. The obtained connectome resembles the results reported in the main text, although with a lower number of connections.

5. DLB and PDD groups

In our work we grouped DLB and PDD participants together and divided them in two groups based on the existence of the visual hallucination feature. For exploratory purpose, we investigated whether any difference between DLB and PDD groups existed in terms of functional connectivity. By performing the NBS analysis, we did not find any significant differential network component. This result is in agreement with our previous publication where we investigated EEG-network properties of different types of dementia in the sensor domain.[6] Demographics of the two diagnostic groups are reported in Table SI.

Table S1 - Demographic data and clinical scores.

Demographic information	DLB (N=18)		PDD (N=24)		Statistics
Age	76.28	±6.64	71.83	±4.99	*P* = 0.023₸
Male/Female	14/4		23/1		*P* = 0.074ⱡ (df=1)
MMSE	23.00	±4.10	24.38	±3.83	*P* = 0.2881₸
Duration of dementia (years)	1.0	±0.7	4.4	±5.4	*P* = 0.016₸
NPI hall total (frequency x severity)	1.56	±1.79	1.42	±1.74	*P* = 0.832₸
UPDRS III	15.50	±8.10	30.96	±15.84	*P* < 0.001₸
AChel (yes/no)	17/1		15/8*		*P* = 0.054ⱡ (df=2)
LEDD	163	±213	822	±432	*P* < 0.001₸

df: degrees of freedom
₸ Unpaired Mann-Whitney U test
ⱡ χ2 test
* One PDD patient was on Memantine.

6. EEG network differences: theta and beta frequency bands

We focused our analyses on the EEG alpha frequency band, since it is reportedly the most linked to lower level visual processing, whilst other frequency ranges such as beta are likely associated with higher level attentional processes.[7] In addition, abnormal alteration of EEG alpha rhythm features has been reported to be associated with Lewy body dementia and to show high specificity in discriminating this syndrome against other forms of dementia and the healthy condition.[8, 9] For completeness, we used the Network Based Statistics (NBS) to test whether any significant differential network component emerged within the other frequency bands; specifically we tested theta (4.6-7 Hz) and beta (15-20 Hz), and no significant components were detected ($P > 0.05$). We also did not find any significant difference in the modularity measure.

7. Alternative measures of structural connectivity

In our work we chose the number of white matter streamlines as a measure of structural connectivity strength between brain regions, as we hypothesized it to be associated with the degree of pathological degeneration. However, this choice comes with some advantages. For instance, it might be biased by the persistence of atrophied white matter fibres. With an exploratory purpose, we measured the fractional anisotropy and mean diffusivity of the fibres connecting the NBM to the NBS-detected cortical regions, as these produced our most significant result when compared between groups and correlated with the functional connectivity strength. Unfortunately, by comparing the white matter properties along the reconstructed tracts between groups and testing whether any correlation existed with functional connectivity strength, we did not find any significant result (Figure SV). It is possible that a higher statistical power, i.e. larger sample size, might lead to the outcome obtained by solely measuring the number of streamlines, although several reasons might influence the correct interpretation of the contrasting results.[10] This should be investigated in future studies.

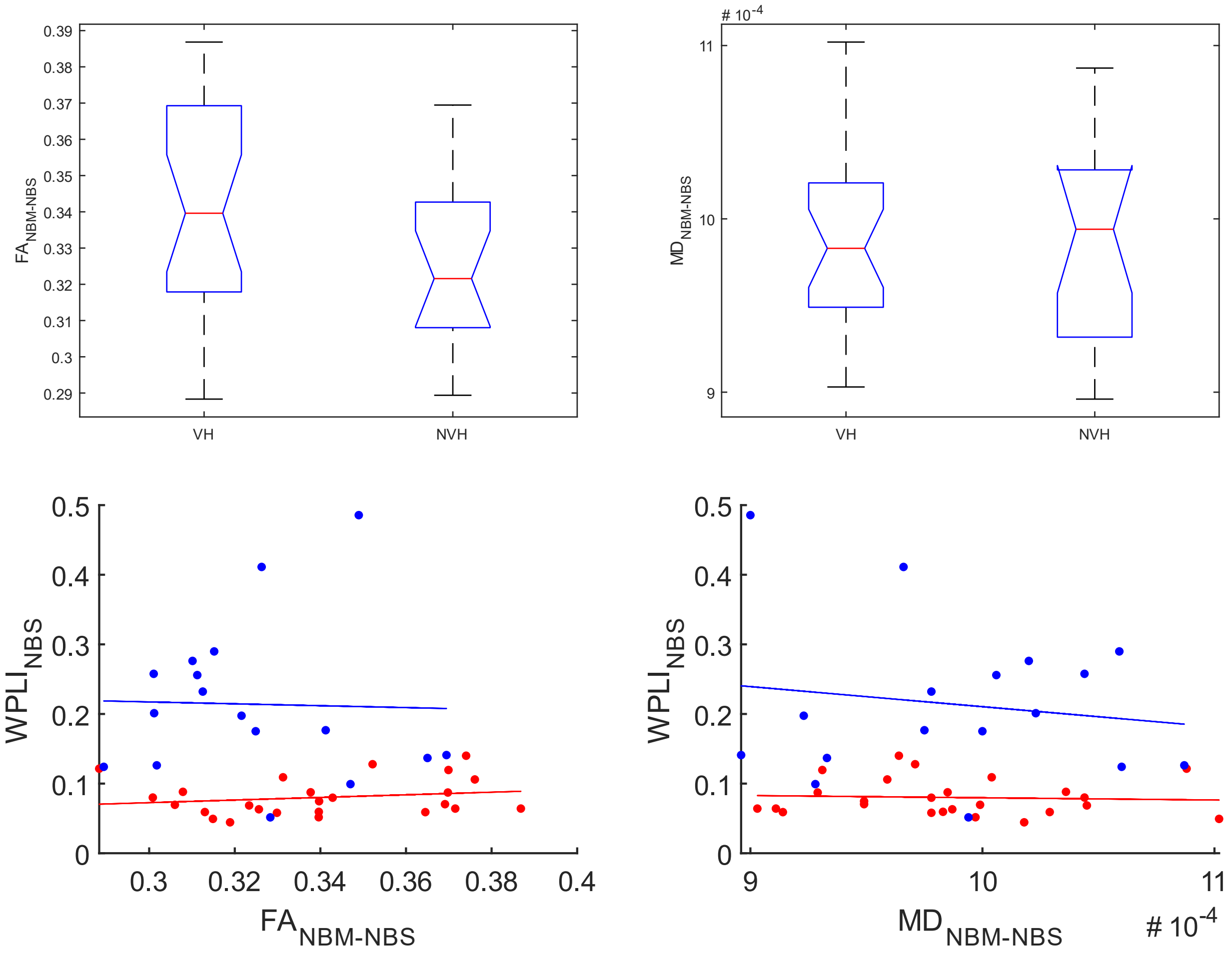

Figure SV – NBM-cortex connectivity: FA and MD. No significant results emerged from the between-group comparison and correlation with EEG connectivity.

8. EEG network coordinates

For visualization purpose, node coordinates were obtained as mass centroids of the ICBM152 head-model[11] vertices within each of the 148 ROIs.[12] Coordinates in the MNI space are reported in Table SII.

Label	**x**	**y**	**z**
'Pole_occipital_L'	-16,856895	-100,1667	-5,6742534
'Pole_occipital_R'	18,1147846	-95,929877	-2,8385904
'G_occipital_sup_L'	-13,980972	-92,103146	30,6393072
'S_oc_middle_and_Lunatus_L'	-31,049869	-89,221256	6,53669457
'G_occipital_sup_R'	18,3290267	-87,948505	35,1847473
'G_occipital_middle_L'	-40,138361	-87,491509	13,4534898
'S_oc_sup_and_transversal_L'	-25,742421	-84,568726	20,9439325
'G_and_S_occipital_inf_L'	-38,461317	-83,414618	-11,446184
'G_cuneus_L'	-3,3468657	-81,646603	17,567933
'S_oc_middle_and_Lunatus_R'	37,2338349	-81,03735	9,27653886
'S_oc_sup_and_transversal_R'	30,1793647	-80,346951	22,1792641
'S_collat_transv_post_L'	-24,259606	-79,935652	-7,833175
'G_occipital_middle_R'	46,3450485	-79,025415	15,2361303
'S_collat_transv_post_R'	27,3908389	-78,88232	-8,8665384
'G_cuneus_R'	5,90665408	-78,013931	19,8297602
'G_oc-temp_med-Lingual_L'	-9,5373903	-74,311454	-5,9839477
'G_and_S_occipital_inf_R'	44,2472872	-74,053108	-12,277754
'S_calcarine_L'	-15,496666	-72,070582	4,71003843
'S_parieto_occipital_L'	-15,504848	-69,375785	25,8156653
'G_pariet_inf-Angular_L'	-45,302616	-68,591351	42,2196655
'S_occipital_ant_L'	-43,101425	-68,519245	4,15231965
'G_oc-temp_med-Lingual_R'	9,37101745	-67,3593	-3,6127028
'S_parieto_occipital_R'	17,3258766	-66,76769	27,3665577
'S_calcarine_R'	17,2170709	-66,715428	6,10398333
'S_occipital_ant_R'	47,6111792	-62,243474	4,33894048
'G_parietal_sup_L'	-23,406961	-61,896369	59,818168
'G_pariet_inf-Angular_R'	49,5164272	-60,790665	42,767234
'G_precuneus_L'	-4,134602	-60,289891	43,6100051
'G_precuneus_R'	4,79367477	-60,271867	46,5138273
'S_intrapariet_and_P_trans_L'	-30,657589	-59,100794	41,7982714
'G_parietal_sup_R'	25,1441688	-57,872513	63,2070373
'S_intrapariet_and_P_trans_R'	30,5651545	-56,858196	44,7500326

'S_interm_prim-Jensen_L'	-52,211148	-55,547456	36,9375096
'G_oc-temp_lat-fusifor_L'	-38,018595	-54,096665	-20,14663
'G_oc-temp_lat-fusifor_R'	34,2769577	-51,637045	-20,75457
'S_oc-temp_lat_L'	-44,815804	-51,018649	-11,938669
'S_oc-temp_lat_R'	43,8580055	-48,834051	-14,267307
'S_subparietal_L'	-9,5142274	-48,27462	35,7512872
'S_subparietal_R'	8,9537357	-48,226819	37,5207114
'G_cingul-Post-ventral_R'	7,09224502	-46,656395	5,58728157
'S_oc-temp_med_and_Lingual_L'	-28,407234	-46,364172	-13,258884
'S_oc-temp_med_and_Lingual_R'	29,0422694	-45,597104	-12,87741
'G_cingul-Post-ventral_L'	-7,6501913	-44,821491	3,14298627
'S_interm_prim-Jensen_R'	53,4322737	-43,416018	35,9316095
'S_temporal_sup_L'	-49,801234	-42,712461	7,05405015
'G_temporal_inf_L'	-55,181424	-40,845183	-21,999078
'G_cingul-Post-dorsal_L'	-2,2440024	-40,328792	30,7762786
'G_temp_sup-Plan_tempo_L'	-60,074589	-39,214686	21,6403281
'S_temporal_sup_R'	50,8477299	-38,19845	9,12947461
'G_cingul-Post-dorsal_R'	1,85359023	-38,108126	33,1704614
'G_temporal_inf_R'	55,1884657	-38,095535	-24,232985
'S_cingul-Marginalis_L'	-13,062979	-36,829735	48,9899833
'G_and_S_paracentral_L'	-7,3626139	-36,226501	69,8499747
'G_pariet_inf-Supramar_L'	-61,114305	-35,991977	33,2433335
'G_and_S_paracentral_R'	7,88003389	-35,652382	71,5312243
'S_cingul-Marginalis_R'	11,9953128	-35,555264	49,2884566
'S_temporal_inf_R'	56,0866664	-34,854645	-14,660639
'Lat_Fis-post_L'	-41,857889	-34,072207	19,4094988
'S_postcentral_L'	-40,476822	-33,284302	46,1508686
'S_temporal_inf_L'	-55,519184	-31,191312	-18,6941
'G_pariet_inf-Supramar_R'	58,3919828	-30,067496	37,0142107
'S_postcentral_R'	39,559387	-29,737032	46,4406676
'G_temporal_middle_L'	-62,091079	-28,564557	-11,401692
'G_temp_sup-Plan_tempo_R'	60,8702299	-28,49692	21,4961563
'Lat_Fis-post_R'	40,1730677	-24,817006	18,5783136
'G_postcentral_L'	-47,507469	-24,046153	55,9848725
'S_temporal_transverse_L'	-53,333727	-22,0591	5,73130557
'G_temporal_middle_R'	62,3925978	-21,611309	-15,007033
'G_postcentral_R'	47,0899879	-21,027259	56,1218101
'G_temp_sup-G_T_transv_L'	-47,5505	-20,574775	9,92095113
'S_central_L'	-38,25244	-20,149708	46,0067886
'S_collat_transv_ant_L'	-42,103808	-19,177564	-28,981915
'S_central_R'	37,3316732	-18,272409	46,4338759
'S_temporal_transverse_R'	52,3678114	-18,024797	9,32791289
'S_collat_transv_ant_R'	41,7743007	-17,738935	-29,330809
'G_oc-temp_med-Parahip_L'	-25,057202	-17,490021	-26,242779
'G_oc-temp_med-Parahip_R'	23,8832041	-13,10857	-27,340384
'G_temp_sup-G_T_transv_R'	48,2852039	-12,302355	7,36303752
'G_and_S_subcentral_L'	-57,601796	-10,807142	14,2693463
'S_precentral-sup-part_L'	-27,848176	-10,494424	56,7640435
'G_Ins_lg_and_S_cent_ins_L'	-41,825645	-10,458351	2,23362653

'G_and_S_cingul-Mid-Post_L'	-6,8746307	-10,239292	41,5425646
'S_circular_insula_inf_L'	-41,121354	-10,133728	-5,6777533
'S_pericallosal_R'	4,30568889	-8,4860049	18,3588585
'G_precentral_L'	-43,837353	-8,4417451	54,910341
'S_precentral-sup-part_R'	28,6509131	-8,3610497	56,5433254
'S_circular_insula_inf_R'	41,2795259	-8,1585978	-6,7111148
'G_and_S_cingul-Mid-Post_R'	6,50004415	-7,7839869	43,8611272
'G_and_S_subcentral_R'	57,5925372	-7,347811	13,2478443
'S_pericallosal_L'	-4,3191537	-6,9543949	19,463515
'G_temp_sup-Lateral_L'	-58,757359	-6,301999	-8,2924862
'G_precentral_R'	43,4064661	-6,0062333	54,1418552
'G_Ins_lg_and_S_cent_ins_R'	41,0476219	-4,1843056	-2,0747366
'G_temp_sup-Lateral_R'	60,0830469	-4,1825595	-6,4375975
'S_precentral-inf-part_L'	-44,575172	4,73598383	30,7636279
'Pole_temporal_L'	-32,059063	5,8561879	-42,534359
'S_circular_insula_sup_L'	-36,382066	6,36621618	11,0907429
'S_precentral-inf-part_R'	43,232989	7,0130399	30,9769796
'G_subcallosal_L'	-9,3007413	7,64565551	-13,67521
'G_insular_short_L'	-41,382944	8,3707236	-3,5490326
'G_subcallosal_R'	3,97513351	8,8112282	-10,74926
'G_temp_sup-Plan_polar_L'	-38,32092	8,93823446	-19,277701
'S_circular_insula_sup_R'	36,0867079	10,1757653	9,78910851
'G_temp_sup-Plan_polar_R'	37,8185339	10,644779	-19,135848
'Pole_temporal_R'	32,0010683	10,8225352	-42,322704
'G_front_inf-Opercular_R'	53,0049681	13,2134486	7,86952158
'G_insular_short_R'	40,1523395	13,2278407	-3,9137817
'G_front_inf-Opercular_L'	-52,695732	13,4175965	10,8084564
'G_and_S_cingul-Mid-Ant_L'	-6,3511835	17,120075	36,7664882
'G_and_S_cingul-Mid-Ant_R'	5,64775196	18,6216281	37,9453958
'Lat_Fis-ant-Vertical_L'	-47,339246	20,4408408	9,84885165
'S_front_sup_L'	-24,146665	22,3512719	44,793897
'S_circular_insula_ant_L'	-31,336991	22,5220224	-8,4050156
'Lat_Fis-ant-Vertical_R'	50,3782686	23,1261706	8,80138268
'S_circular_insula_ant_R'	31,7375088	23,9660666	-8,2205039
'S_orbital_med-olfact_L'	-12,789413	26,2275148	-20,002537
'S_orbital_med-olfact_R'	11,3397309	26,5968279	-19,801781
'S_front_inf_L'	-40,559908	26,8651178	19,8092285
'S_front_sup_R'	23,9212788	26,8800996	43,9376926
'G_front_sup_L'	-9,8095551	27,1560262	46,6208742
'G_front_inf-Orbital_L'	-48,374082	27,7471761	-10,024611
'G_front_sup_R'	9,54122583	28,6498217	46,1859897
'S_front_inf_R'	40,022604	29,0828579	20,520266
'Lat_Fis-ant-Horizont_R'	40,7323844	29,6613769	-1,9917581
'G_front_inf-Triangul_L'	-52,34446	30,9573826	2,63543048
'Lat_Fis-ant-Horizont_L'	-43,401756	32,2568612	-4,6294391
'G_rectus_R'	2,87156024	32,3256205	-25,355636
'G_front_inf-Triangul_R'	53,2683917	32,6627652	5,90740657
'G_orbital_L'	-31,042782	34,1044698	-20,590706
'G_orbital_R'	29,1962396	35,6463052	-20,406356

'G_front_middle_L'	-38,591993	35,7733403	33,0580822
'G_front_inf-Orbital_R'	51,1508807	36,9698238	-13,594139
'S_orbital-H_Shaped_L'	-27,354868	37,6675795	-16,42051
'G_rectus_L'	-4,1211068	38,1418317	-23,422932
'G_front_middle_R'	38,6936979	38,2369656	32,078083
'S_orbital-H_Shaped_R'	26,8075877	38,7712856	-16,740152
'S_suborbital_R'	3,15998605	41,9296244	-16,421215
'G_and_S_cingul-Ant_R'	5,85787713	42,3592462	4,55362515
'G_and_S_cingul-Ant_L'	-7,2514034	42,3919445	6,63595362
'S_suborbital_L'	-5,220484	43,9476634	-12,708837
'S_orbital_lateral_L'	-43,648027	45,4283591	-5,5656196
'S_orbital_lateral_R'	43,6923782	46,5803129	-3,1078501
'S_front_middle_L'	-28,307357	47,0646389	19,3032746
'S_front_middle_R'	28,9039738	47,4571278	17,8662803
'G_and_S_frontomargin_L'	-25,310918	60,9520505	-9,0834118
'G_and_S_frontomargin_R'	21,2763927	63,2872608	-11,452679
'G_and_S_transv_frontopol_L'	-16,838447	68,2448486	-2,3301895
'G_and_S_transv_frontopol_R'	17,4681568	68,4477277	0,18827672

Table S11 - Network node coordinates based on the Destrieux atlas[12] in the MNI space. Nodes are computed as mass centroids within each parcellated region.

References

1. Graziadio S, Basu A, Tomasevic L, Zappasodi F, Tecchio F, Eyre JA. Developmental tuning and decay in senescence of oscillations linking the corticospinal system. *J Neurosci*. Mar 10 2010;30(10):3663-74. doi:10.1523/jneurosci.5621-09.2010

2. Pfurtscheller G. Spatiotemporal ERD/ERS patterns during voluntary movement and motor imagery. *Supplements to Clinical neurophysiology*. Elsevier; 2000:196-198.

3. Nelson AB, Moisello C, Lin J, et al. Beta oscillatory changes and retention of motor skills during practice in healthy subjects and in patients with Parkinson's disease. *Frontiers in human neuroscience*. 2017;11:104.

4. Ricci S, Mehraram R, Tatti E, et al. Aging Does Not Affect Beta Modulation during Reaching Movements. *Neural plasticity*. 2019;2019

5. Tatti E, Ricci S, Mehraram R, et al. Beta Modulation Depth Is Not Linked to Movement Features. Brief Research Report. *Frontiers in Behavioral Neuroscience*. 2019-March-14 2019;13(49)doi:10.3389/fnbeh.2019.00049

6. Mehraram R, Kaiser M, Cromarty R, et al. Weighted network measures reveal differences between dementia types: An EEG study. *Human brain mapping*. Dec 9 2019;doi:10.1002/hbm.24896

7. Kopell N, Ermentrout GB, Whittington MA, Traub RD. Gamma rhythms and beta rhythms have different synchronization properties. *Proceedings of the National Academy of Sciences*. 2000;97(4):1867-1872. doi:10.1073/pnas.97.4.1867

8. Briel RC, McKeith IG, Barker WA, et al. EEG findings in dementia with Lewy bodies and Alzheimer's disease. *J Neurol Neurosurg Psychiatry*. Mar 1999;66(3):401-3.

9. Peraza LR, Cromarty R, Kobeleva X, et al. Electroencephalographic derived network differences in Lewy body dementia compared to Alzheimer's disease patients. *Sci Rep*. Mar 15 2018;8(1):4637. doi:10.1038/s41598-018-22984-5

10. Jones DK, Knösche TR, Turner R. White matter integrity, fiber count, and other fallacies: The do's and don'ts of diffusion MRI. *NeuroImage*. 2013/06/01/ 2013;73:239-254. doi:https://doi.org/10.1016/j.neuroimage.2012.06.081

11. Tadel F, Baillet S, Mosher JC, Pantazis D, Leahy RM. Brainstorm: a user-friendly application for MEG/EEG analysis. *Computational intelligence and neuroscience*. 2011;2011:879716. doi:10.1155/2011/879716

12. Destrieux C, Fischl B, Dale A, Halgren E. Automatic parcellation of human cortical gyri and sulci using standard anatomical nomenclature. *NeuroImage*. 2010;53(1):1-15. doi:10.1016/j.neuroimage.2010.06.010
